# Electrospun poly(vinyl alcohol) nanofibers loaded with quaternary ammonium compounds for localized eradication of periodontal biofilms

**DOI:** 10.1039/d6ma00633g

**Published:** 2026-09-03

**Authors:** Ting Chen, Nicola Kelly, Pedro Leonidas Oseliero Filho, Megan Carr, Alexander Ciupa, Jenny Aveyard, Raechelle A. D'Sa

**Affiliations:** a School of Engineering, University of Liverpool, Brownlow Hill Liverpool L69 3GH UK r.dsa@liverpool.ac.uk; b Materials Innovation Factory, University of Liverpool, Oxford Street Liverpool L7 3NY UK

## Abstract

Periodontitis is driven by polymicrobial biofilms and remains difficult to treat using systemic antibiotics, which fail to achieve therapeutic concentrations within periodontal pockets. In this study, we report on the fabrication of electrospun poly(vinyl alcohol) (PVA) nanofibers incorporating quaternary ammonium compounds (QACs)-specifically benzyltriethylammonium chloride (BTEAC) and benzyldimethyldodecylammonium chloride (BDMDAC) as localized antimicrobial platforms. Electrospinning parameters were optimized to achieve high drug loading and reproducible fiber morphologies. Incorporation of QACs reduced their porosity and surface area, while thermal stability increased because of enhanced intermolecular interactions. The nanofibers exhibited high wettability and sufficient integrity for specimen handling for oral applications, and the structures remained stable in artificial saliva. Both formulations demonstrated rapid *in vitro* drug release. Antimicrobial testing against both planktonic and biofilm forms of *Staphylococcus aureus*, *Escherichia coli*, *Streptococcus mutans*, and *Porphyromonas gingivalis*, revealed structure, dose and environment-dependent efficacy. PVA-BTEAC showed pronounced bactericidal activity against *E. coli*, *S. mutans*, *and P. gingivalis*, whereas PVA-BDMDAC exhibited strong bactericidal effects against *S. aureus*, *S. mutans*, and *P. gingivalis*. Most QAC loadings were non-cytotoxic to L929 fibroblasts; however, the highest dose of BTEAC was cytotoxic, consistent with a burst-release profile. Overall, these results demonstrate that QAC-loaded PVA nanofibers exhibit rapid and complete antimicrobial agent release, alongside broad-spectrum efficacy against both planktonic and biofilm-associated bacteria, supporting their potential as localized intraoral delivery platforms for periodontal biofilm eradication. BDMDAC-loaded nanofibers in particular show promise for application in the dynamic environment of periodontal pockets.

## Abstract 2

Quaternary ammonium compound-loaded PVA nanofibres combine antimicrobial release, potent antibiofilm activity, and favourable physicochemical performance, highlighting their promise as fast-acting local inserts following periodontal debridement.

## Graphical abstract

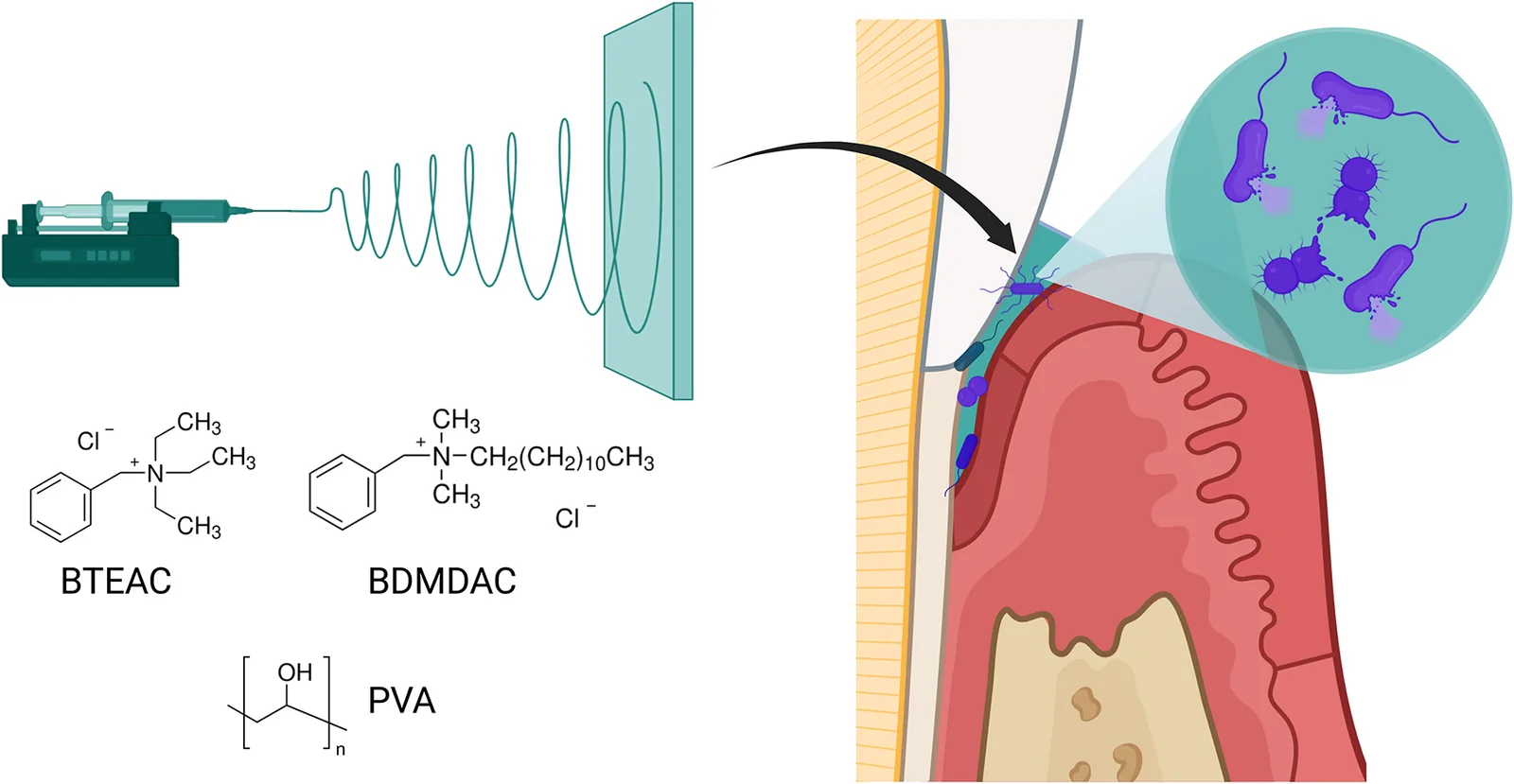

## 1. Introduction

The World Health Organization (WHO) estimates nearly 3.5 billion people worldwide suffer from oral diseases, with periodontal disease posing a significant burden. This condition affects roughly 19% of the global adult population, amounting to more than 1 billion cases.^1^ The disease is initiated by polymicrobial biofilms that colonise the tooth surface and periodontal pockets, leading to chronic inflammation and progressive destruction of the gingiva, periodontal ligament, alveolar bone, and root surfaces. The warm, moist environment of the oral cavity promotes microbial proliferation, while the biofilm matrix confers increased resistance to antimicrobial agents and mechanical removal.^2^ If left untreated, chronic periodontitis can result in pain, inflammation, tooth loss, and eventually systemic complications such as sepsis.^3^

The standard treatment for periodontitis typically involves non-surgical interventions such as debridement and other mechanical methods combined with systemically administered antibiotics.^3,4^ However, systemic antibiotics rarely achieve therapeutic concentrations within the periodontal pockets and may promote off-target toxicity and the development of antibiotic resistance. Localized delivery of antimicrobials, therefore, represents an attractive strategy that can reduce the risk of developing antibiotic resistance and combat any off-site toxicity issues.^5^

Quaternary ammonium compounds (QACs) are promising alternatives to antibiotics due to their broad-spectrum antimicrobial activity, their ability to effectively penetrate and disrupt biofilms, and their low toxicity to human cells at therapeutic concentrations.^6^ QACs interact through electrostatic interactions, which can disrupt the negatively charged bacterial cell wall through a contact killing mechanism. The positively charged end or “head” of the QAC interacts with the polar surface of the membrane by displacing stabilizing Ca^2+^ and Mg^2+^ cations. The hydrophobic end or “tail” is then inserted into the phospholipid bilayer, causing the membrane to lose its structural integrity, resulting in cytoplasmic leakage and cell death. Since their introduction into dentistry, QACs have been incorporated into dental resins, fillers, and coatings to improve antimicrobial performance and infection control.^7–10^ Whilst QACs are effective broad-spectrum antimicrobials, their potential to select for resistant strains through efflux pump upregulation at sub-inhibitory concentrations represents a recognized risk that must be considered in the design of QAC-based delivery systems.

Localized intra-pocket delivery of QACs offers a strategy to control periodontal infection by concentrating therapeutic antimicrobial agents directly at the disease site.^11^ Such delivery systems must provide effective antimicrobial concentrations at the target site, degrade into non-toxic by-products and ideally support periodontal regeneration. Electrospinning represents a promising manufacturing technique for intra-pocket drug delivery and has been widely applied in the development of nanofibrous drug delivery systems. This technique fabricates ultra-fine fibers from polymer solutions or melts, yielding materials with high surface area-to-volume ratios, tunable porosity, and the capacity to incorporate a variety of bioactive agents.^12,13^ The large surface area inherent to electrospun architectures facilitates rapid and complete release of incorporated antimicrobials, which may be particularly advantageous in periodontal applications where immediate disruption of established biofilms is the primary therapeutic objective. These electrospun fibers can be tailored to create periodontal patches or membranes loaded with antimicrobials, anti-inflammatory agents, or growth factors, delivering therapeutic agents directly at the target site. For example, Joshi *et al.* have developed PVA/hyaluronic acid (HA) nanofibers exhibiting good adhesive properties and antimicrobial efficacy, showing potential for antimicrobial and restorative efficacy.^14^ Bottino *et al.* developed biodegradable metronidazole- and ciprofloxacin-loaded electrospun polymer nanofibers and demonstrated direct inhibition of key periodontopathogens including *Fusobacterium nucleatum*, *Aggregatibacter actinomycetemcomitans*, and *Porphyromonas gingivalis*, establishing nanofibrous scaffolds as an efficacious platform for subgingival antimicrobial delivery.^15^

Periodontal infections are among the most prevalent chronic oral diseases and are driven by complex microbial communities residing in the oral cavity. The human oral microbiome comprises over 800 microbial taxa, and the diversity and interspecies interactions within these communities contribute to the persistence of periodontal biofilms and their resilience to host defenses and antimicrobial intervention.^3,16^ Given the complexity and persistence of these communities, there is a growing need for effective antimicrobial therapies that manage periodontal disease while preserving the wider oral microbiome. In this study, we report on the fabrication, characterization and biological evaluation of electrospun PVA/QAC patches for the treatment of periodontal infections. PVA is a synthetic water-soluble polymer with excellent biocompatibility, processability, mechanical durability, and biodegradability.^17,18^ PVA has been extensively used for various biomedical applications.^12,19,20^ It is generally recognized as safe (GRAS) by the US Food and Drug Administration (FDA) and does not have any immunogenic effects, with demonstrated efficacy in long-term oral, topical and ophthalmic applications.^21–23^ The PVA nanofibers were loaded with two types of QACs: benzyltriethylammonium chloride (BTEAC) and benzyldimethyldodecylammonium chloride (BDMDAC). These two QACs were chosen because they both contain a common benzyl quaternary ammonium headgroup. This allows for a direct comparison of the cationic “active” center. However, they differ significantly in their alkyl substituents: BTEAC has short ethyl groups, while BDMDAC features a long C12 chain. This difference is expected to influence their hydrophobicity, interactions with membranes, and thus their antimicrobial effectiveness, cytocompatibility, and release behaviour.^24^ Nanofiber fabrication was optimized, and QAC loading systematically varied to identify formulations with optimal antimicrobial performance. The mechanical properties, wettability, chemical composition, and drug release profiles were characterized. Antimicrobial activity was evaluated against planktonic and biofilm-adhered states of Gram-positive (*Staphylococcus aureus and Streptococcus mutans*) and Gram-negative (*Escherichia coli* and *Porphyromonas gingivalis*) bacteria. The biocompatibility was assessed using L929 fibroblast cells.

## 2. Materials and methods

### 2.1 Materials

Poly(vinyl alcohol) (PVA) (M_w_ = 85 000–124 000, 99% hydrolyzed), benzyltriethylammonium chloride (BTEAC), benzyldimethyldodecylammonium chloride (BDMDAC), Luria-Bertani (LB) broth and agar, Columbia broth (CB) and agar, Dulbecco's modified Eagle's medium (DMEM), penicillin and streptomycin (PS), and trypsin were acquired from Merck (Gillingham, UK) and used as received. Bacto™ Tryptic soy broth (TSB) and agar were acquired from Fisher Scientific (Leicestershire, UK). Mouse fibroblast cells L929 were purchased from Lonza Bioscience (Slough, UK). 3-(4,5-Dimethylthiazol-2-yl)-2,5-diphenyltetrazolium bromide (MTT) powder, dimethyl sulfoxide (DMSO) all purchased from Thermo Fisher (Loughborough, UK). Sheep blood was purchased from Darwin Biological (Shropshire, UK). Fetal bovine serum (FBS) was acquired from Biosera (Nuaille, France). All common, analytical grade laboratory solvents and salts, including ethanol, hydrochloric acid (HCl), sodium chloride (NaCl), and disodium phosphate (Na_2_HPO_4_), sodium carboxymethyl cellulose (CMC), potassium chloride (KCl), magnesium chloride hexahydrate (MgCl_2_·6H_2_O), calcium chloride dihydrate (CaCl_2_·2H_2_O), dipotassium phosphate (K_2_HPO_4_), potassium dihydrogen phosphate (KH_2_PO_4_), menadione, hemin, methyl-*p*-hydroxybenzoate were from Sigma-Aldrich (St. Louis, MO, USA).

### 2.2 Solution preparation and solution properties

The polymer solutions of PVA were prepared by dissolving 8 wt%, 9 wt% and 10 wt% of PVA powder in deionized water at 90 °C with vigorous stirring for 4 hours. Subsequently, 3 wt% and 6 wt% of BTEAC and 0.01 wt% and 0.1 wt% of BDMDAC were added to the PVA solution individually. The mixtures were stirred until homogeneous solutions were formed, ensuring proper dispersion of the additives within the PVA matrix for electrospinning.

The viscosity of polymer solutions was examined by a rotary viscometer (NDJ-1, Shanghai, China). 30 mL of the test solutions were poured into a 30 mL beaker with an inner diameter of 35 mm, and a length of 56 mm. Rotor #1 was selected, and the viscosity was measured after stirring for 1 min at a rotational speed of 12 rpm. The conductivity of solutions was measured by a conductivity meter (RS 1410, Northamptonshire, UK) at room temperature. 30 mL of test solutions were prepared in the same way as the previous method. A measuring probe was placed in the solution for 30 seconds to obtain stable reading. The surface tension of polymer solutions was measured by a pendant drop method using Attension ThetaLite and OneAttension software (Biolin Scientific, Västra Frölunda, Sweden). A 5 µL pendant drop of the polymer solution was hanging from the needle tip, and with the software, where the shape of the drop was captured for 10 seconds, the surface tension of the sample was measured. These experiments were performed in triplicate.

### 2.3 Electrospinning

Electrospinning of the polymer solutions was performed using a climate-controlled electrospinner (IME, Waalre, Netherlands). The solutions were transferred into a 3 mL plastic syringe fitted with a 0.4 mm blunt-ended metallic needle. A cylindrical grounded collector (90 mm × 180 mm) was coated with aluminum foil. Applied voltages were varied between 16 kV and 24 kV; flow rates were varied between 0.4 mL h^−1^ and 1.5 mL h^−1^; distances between syringe and collector were varied between 11 cm and 19 cm; processing temperatures were varied between 23 °C and 25 °C, and processing humidity varied between 30% and 50%. These parameters were used for spinning for 5 minutes for SEM imaging. The optimal parameters were applied for collecting uniform nanofibers. Following 6 hours of collection at a 60 mm scanning distance and 50 rpm, the nanofibers were stored in a desiccator to remove residual solvent overnight from the solution.

### 2.4 Morphology of electrospun PVA and PVA-QAC nanofibers

The morphology of three types of nanofibers, namely PVA nanofibers, PVA-BTEAC nanofibers, and PVA-BDMDAC nanofibers, was examined using SEM. Before imaging, the samples were coated with a thin layer of gold using a sputter coater (Q150T ES, East Sussex, UK). Images were captured using a JSM-7001F field-emission scanning electron microscope (Tokyo, Japan) at 15 kV. To determine the fiber diameter, ImageJ 1.48 software was utilized. A total of 200 individual fibers were measured from each collected sample for subsequent analysis of the fiber diameter.

### 2.5 Characterization of nanofibers

#### 2.5.1 Apparent density and porosity

The thickness of electrospun PVA nanofibers, PVA-BTEAC and PVA-BDMDAC nanofibers was measured by micrometer. Triplicate measurements were performed, and their apparent density and total porosity were calculated by the following formula:^25^1
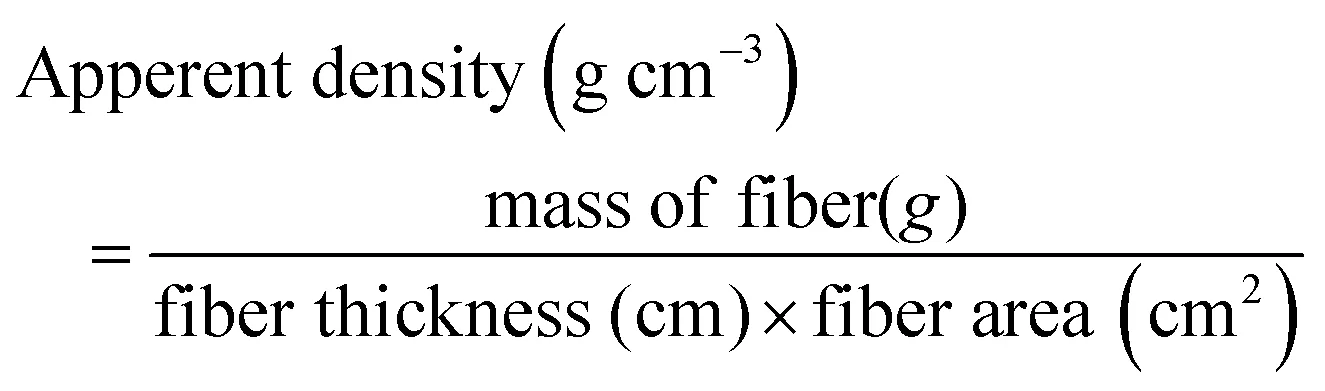
2
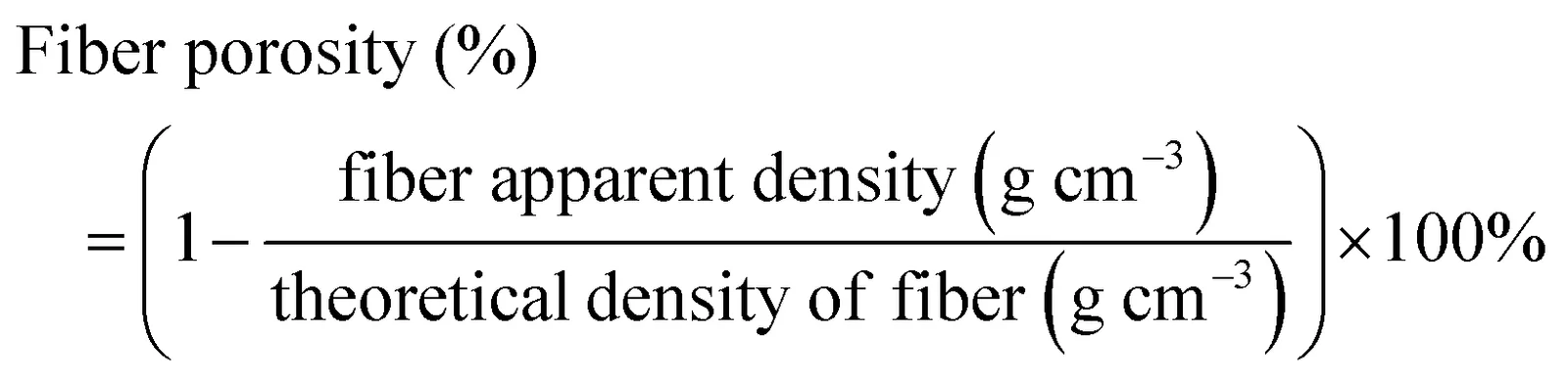


#### 2.5.2 Surface area

The surface area and pore size of electrospun PVA nanofibers, PVA-BTEAC and PVA-BDMDAC nanofibers were measured using a 3Flex high-performance gas adsorption analyzer (Micromeritics, USA) at 77 K in nitrogen. Samples (100 ± 10 mg) were loaded in a 12 mm flat-bottom sample tube. Before the measurement, samples were degassed in a mantle for 4 h. The relative pressure range P/P0 of 0.05–0.25 was used for calculating the surface area with a Micromeritics-3Flex analyzer, employing the Brunauer–Emmett–Teller (BET). Micropore analysis was performed using the Horvath–Kawazoe (HK) method, while pore size distribution and mesopore characterization were determined by the Barrett–Joyner–Halenda (BJH) method applied to the desorption branch of the isotherm.

#### 2.5.3 Contact angle

The static contact angles of the samples were measured using the sessile drop method, employing Attension ThetaLite and OneAttension software (Biolin Scientific, Västra Frölunda, Sweden). Upon contact of the droplet with the sample surface, a 10-second video was recorded, and the contact angle was extracted from the first frame of the recording to represent the initial wetting behavior at *t* = 0 s. Triplicate measurements were performed for each sample.

#### 2.5.4 Tensile testing

The mechanical properties of the samples were assessed through tensile testing in the dry state, following the methodology described in a prior publication.^25^ The specimens were cut into a rectangular shape measuring 3 cm × 1 cm, with the final testing dimensions set at 2 cm × 1 cm. Before subjecting the samples to the testing apparatus, their thickness was measured using an electronic caliper with a precision of 0.01 mm. Tensile testing was performed using a UniVert mechanical tester (CellScale, Waterloo, Ontario, Canada) in tensile mode, with a preload of 0.1 N applied prior to ramping, and a crosshead displacement rate of 10 mm min^−1^. A total of three measurements were conducted per sample.

### 2.6 Fourier transform infrared spectroscopy (FTIR)

To characterize the chemical properties of electrospun nanofibers, a PerkinElmer Frontier IR system (PerkinElmer Inc., Shelton, CT, USA) over the range of 500–4000 cm^−1^ with 64 scans at a scanning resolution of 4 cm^−1^ was utilized.

### 2.7 Thermal stability

The thermal stability of the electrospun nanofibers was assessed *via* thermogravimetric analysis (TGA) (Netzsch TG 209 F1 Libra, Germany) and differential scanning calorimetry (DSC) (TA instruments, USA). A small mass of each sample (1.1–1.6 mg) was placed into an alumina crucible and assessed over a 25–600 °C range, using a heating rate of 10 °C min^−1^, under a 20 mL min^−1^ flow of nitrogen. After the TGA assessment, all samples were assessed for DSC analysis. An accurate mass of each sample was added to a TZero ® Pan (TA instruments, USA) with pin hole lid. Samples of PVA and PVA-BDMDAC fibers were submitted over a 0–200 °C range. A sample of PVA-BTEAC fibers was submitted over a 0–170 °C range, due to the derivative thermogravimetry (DTG) trace from TGA assessment, revealing that the sample started decomposing at around 180 °C. Software including NETZSCH Proteus® and TA Instruments universal analysis Trios was used for data analysis.

### 2.8 QAC release study

Samples were placed on a 12-well plate immersed in 2 mL deionized water on the rocker shaker at room temperature. At 0, 5, 10, and 15 min, 2 mL solution was taken out of the buffer into a quartz cuvette. Then another 2 mL of fresh deionized water was added to each well. The amount of QAC released was determined by UV-vis spectroscopy at a wavelength of 261 nm. The release experiments were performed in triplicate.

### 2.9 Swelling ratio and degradation

Samples were cut into a 10 ± 1 mg squares. The swelling behavior of the nanofibers was determined by immersing the samples in phosphate-buffered saline (PBS) or artificial saliva at 37 °C. Measurements were taken, in triplicate, every day until day 5. The swelling ratio of nanofibers was calculated with the following formula:^26^3
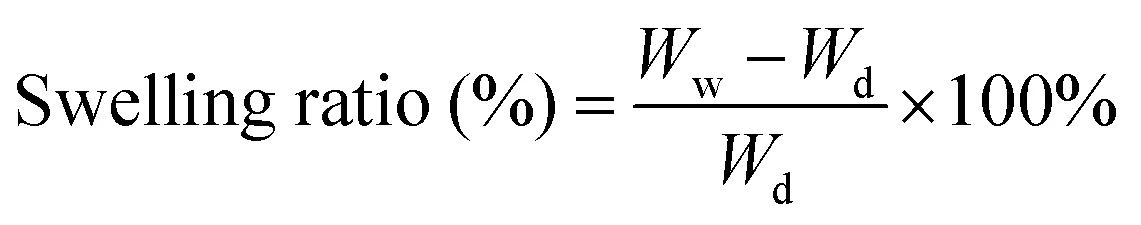
where *W*_w_ and *W*_d_ denote the weight of the sample in the swollen and dry state.

To determine the degradation rate of the PVA and QAC-loaded fibers, samples were immersed in PBS or artificial saliva at 37 °C. The samples were then removed at days 1, 2, 3, 4 and 5, oven-dried overnight and weighed. The remaining weight of the samples was calculated with the following formula:^26^4
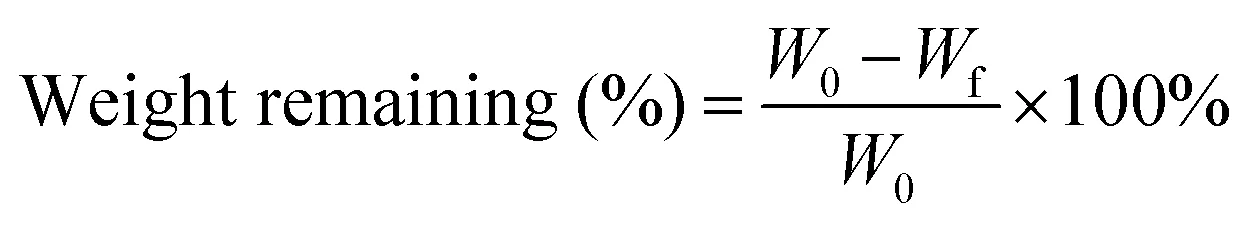
where *W*_0_ and *W*_f_ denote the weight of the sample before the degradation test and after oven drying. Measurements were done in triplicate.

### 2.10 Antimicrobial assays

#### 2.10.1 Minimum inhibitory concentration and minimum bactericidal concentration assay

The minimum inhibitory concentration (MIC) refers to the concentration of antimicrobial agent that inhibits the growth of bacteria, while the minimum bactericidal concentration (MBC) refers to the concentration at which no bacterial survival is observed. The MIC and MBC of BTEAC and BDMDAC were determined against the reference strains *Staphylococcus aureus* (*S. aureus*) National Collection of Type Cultures (NCTC) 13811 and *Escherichia coli* (*E. coli*) NCTC 9001, both sourced from authenticated national culture collections to serve as standardized reference strains for antimicrobial susceptibility assessment, to provide standardized and reproducible benchmarks for antimicrobial susceptibility assessment consistent with established microbiological practice. BTEAC and BDMDAC were dissolved in LB broth, yielding concentrations ranging from 2 mg mL^−1^ to 50 mg mL^−1^ and 0.512 mg mL^−1^ to 0.001 mg mL^−1^, respectively. To prepare the bacterial cultures, overnight cultures of *S. aureus* and *E. coli* in LB broth were diluted to a concentration of 1 × 10^6^ CFU mL^−1^, using a 0.5 McFarland standard.^27^ Subsequently, 0.5 mL of each BTEAC concentration and 0.5 mL of bacteria solution were added into each well, followed by incubating for 24 hours at 37 °C. The absence of visible microbial growth in the wells was assessed as MIC. The lowest concentration at which no bacterial growth was observed corresponded to the MBC. All experiments were repeated three times with three technical replicates.

#### 2.10.2 Antimicrobial activity against planktonic bacteria

Reference strains included *S. aureus* NCTC 13811 and *E. coli* NCTC 9001; dental-specific pathogens included *Streptococcus mutans* (*S. mutans*) NCTC 10449 and *Porphyromonas gingivalis* (*P. gingivalis*) NCTC 11834, which were used to evaluate the antibacterial efficacy of the electrospun nanofibers. PVA nanofibers were used as control samples and four different weights of PVA-BTEAC nanofibers (5, 20, 40, 60 mg corresponding to 1, 5, 10 and 15 mg mL^−1^ of BTEAC in the broth) and PVA-BDMDAC nanofibers (0.9, 4.5, 9, 13.5 mg that corresponding to 0.001, 0.005, 0.010 and 0.015 mg mL^−1^ of BDMDAC in the broth) as testing samples. The overnight cultures of *S. aureus* and *E. coli* were diluted to 1 × 10^6^ CFU mL^−1^ in LB broth, *S. mutans* and *P. gingivalis* were diluted in CB and TSB supplemented with 5 µg mL^−1^ hemin and 1 µg mL^−1^ menadione, respectively. The nanofibers were placed in a 24-well plate, and 1 mL of diluted bacterial solution was added to each well and incubated at 37 °C to allow bacterial growth for 4 hours and 24 hours. At these time points, the solutions from the wells were transferred to a sterile plate and serially diluted with the corresponding broth. The bacterial CFU of incubating solutions were determined *via* the Miles and Misra method on LB agar plates, Columbia agar with 10% sheep blood and Tryptic soy agar with 5 µg mL^−1^ hemin, 1 µg mL^−1^ menadione and 10% sheep blood.^28^ All experiments were repeated three times with three technical replicates.

#### 2.10.3 Biofilm colony forming unit assay on adhered bacteria

Nanofiber samples were placed in a 24-well plate, and 1 mL of diluted bacterial solution (1 × 10^6^ CFU mL^−1^) was added to each well. The plate was incubated for 24 hours at 37 °C. At the 24 hour timepoint, the samples were gently washed with PBS twice to remove planktonic bacteria and then resuspended in 1 mL of the corresponding broth. Samples were then sonicated for 20 minutes to remove attached biofilms. The bacterial CFU of incubating solutions were determined on corresponding agar plates. All experiments were repeated three times with three technical replicates.

### 2.11 Cell viability

The biocompatibility of the QAC-loaded PVA nanofibers was evaluated by leachate cell viability assay with an immortalized murine fibroblast cell line L929. L929 fibroblasts were cultured at 37 °C and 5% CO_2_ in DMEM supplemented with 10% FBS and 1% PS for 24 hours.

Leachate from electrospun nanofibers with QACs and controls was prepared by incubating the materials as per the preparation in antimicrobial assays in 1 mL of supplemented cell culture media for 24 hours at 37 °C. L929 fibroblasts were seeded in 96-well plates at 1 × 10^4^ cells per well and incubated for 24 hours to have an 85–90% confluency rate, after which growth media were removed from wells, and 100 µL leachate and controls were added to each well. Cells were incubated for a further 4 hours and 24 hours. A 1 mg mL^−1^ MTT/PBS solution was prepared. After the 4 hours and 24 hours incubation, all testing wells were aspirated and washed three times with PBS; then, 100 µL of MTT solution was added to the wells. After 4 hours of incubation, the MTT solution was aspirated, and 100 µL of DMSO was added, with mixing on a Stuart gyro-rocker SSL3 for 10 minutes. Cell viability was determined using a Synergy HT microplate reader (BioTek Co., USA) at OD_570_ and OD_630_ and the cell count was determined. Percentage viability was calculated using the equation below, where sample_570mm_ and sample_630mm_ are the UV absorbance of samples at 570 nm and 630 nm, and control_570mm_ and control_630mm_ are the UV absorbance of the control. According to ISO10093-5, materials that demonstrate more than 70% viability are considered noncytotoxic.^29^5
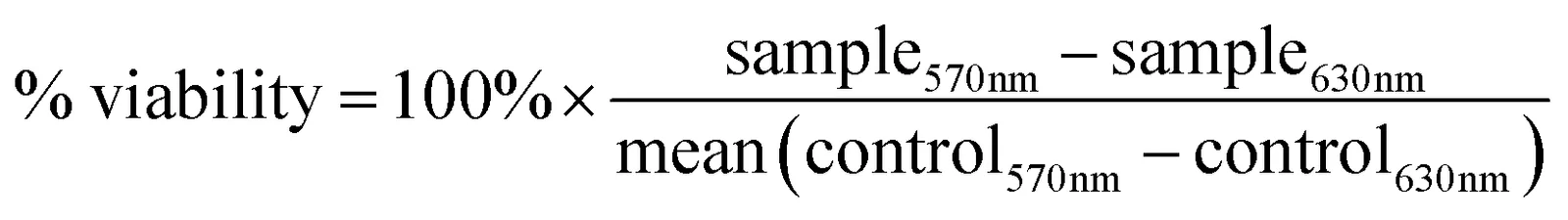
The experiment was repeated three times with six technical replicates.

### 2.12 Statistical analysis

One-way analysis of variance (ANOVA) with the Tukey method was used to analyze the differences among the variously treated samples. Data was collected in triplicate and displayed as mean ± standard deviation. The value of *p* < 0.05 was taken as being statistically significant.

## 3. Results and discussion

### 3.1 Optimization of PVA nanofibers

Electrospinning is a technique that enables the stretching of polymer jets into nanoscale fibers from polymer droplets. However, the successful formation of continuous fibers depends on the application of a sufficient voltage, which stretches the polymer jet to generate a Taylor cone and subsequently form nanofibers. To investigate the effects of processing parameters on the morphology and diameter of PVA nanofibers, various polymer concentrations (8–10%), needle-to-collector distances (11–19 cm), applied voltages (16–24 kV), flow rates (0.4–1.5 mL h^−1^), and relative humidities (30–50%) were examined. The influence of each processing parameter is presented in the SI (Fig. S1–S5).

Smooth and uniformly distributed nanofibers were successfully produced with a PVA concentration of 9%, resulting in an average fiber diameter of 390.6 ± 60.1 nm. Under these conditions, the electrospinning process was more stable and yielded more homogeneous nanofibers. As polymer concentration increased, both the viscosity and the average fiber diameter also increased (Table S1). This trend can be attributed to the strong intermolecular and intramolecular hydrogen bonding between the polar –OH groups in PVA. These interactions enhance chain entanglement and promote gel formation, which in turn increases the solution viscosity. Therefore, the optimal electrospinning parameters were determined to be a polymer concentration of 9%, an applied voltage of 22 kV, a flow rate of 0.8 mL h^−1^, a needle-to-collector distance of 15 cm, and a relative humidity of 50%. These conditions resulted in PVA nanofibers with an average diameter of 390.6 ± 60.1 nm.

### 3.2 Electrospun BTEAC-loaded and BDMDAC-loaded nanofibers

To impart antimicrobial efficacy to the PVA nanofibers, BTEAC and BDMDAC were incorporated to produce QAC-loaded PVA nanofibers. The QAC concentrations used were based on the MIC and MBC values identified for *S. aureus* and *E. coli*. This approach helps establish a standardized baseline for assessing antimicrobial susceptibility, as illustrated in Table 1. The liquid-phase MIC and MBC values serve as valuable benchmarks for evaluating the efficacy of the QAC-loaded fibers in relation to the specific organisms and their respective concentrations. 3% and 6% BTEAC, and 0.01% and 0.1% BDMDAC were incorporated into 9% PVA solution, with their solution properties shown in Table S1. The morphology of the electrospun PVA-BTEAC and PVA-BDMDAC nanofibers and their average fiber diameter at various concentrations are given in Fig. 1. Compared to pure PVA nanofibers, the addition of BTEAC increases the fiber diameter for 3% and 6% BTEAC samples to 469 ± 83 nm and 592 ± 150 nm. The electrospinning process of 3% BTEAC was the most stable at that rate. In addition, compared to the 9% PVA solution that exhibited a viscosity of 234 ± 19 cP, the addition of BTEAC resulted in reduced viscosity, showing 192.7 ± 7.9 cP for 3% and 197.2 ± 2.1 cP for 6%. The incorporation of BTEAC significantly increased the conductivity, with 0%, 3% and 6% PVA-BTEAC showing an increasing conductivity from 1.7 ± 0.1 mS cm^−1^ to 14.2 ± 0.4 mS cm^−1^. And the surface tension of these solutions was 52 ± 1.5 mN m^−1^, 52 ± 0.7 mN m^−1^, and 47 ± 0.3 mN m^−1^, respectively. During electrospinning, the solution containing 3% BTEAC exhibited the highest process stability, whereas increased instability and reduced fiber yield were observed at 6% BTEAC. Therefore, the 3% BTEAC concentration was selected for further investigation.

**Table 1:** MIC and MBC of liquid phase QACs against *S. aureus* and *E. coli*

	BTEAC concentration (mg mL^−1^)		BDMDAC concentration (mg mL^−1^)	
	MIC	MBC	MIC	MBC
*S. aureus*	16.4 ± 4.8	40 ± 5	0.0040 ± 0.0000	0.010 ± 0.004
*E. coli*	10 ± 0	25 ± 0	0.0320 ± 0.0000	0.040 ± 0.016

**Fig. 1:**
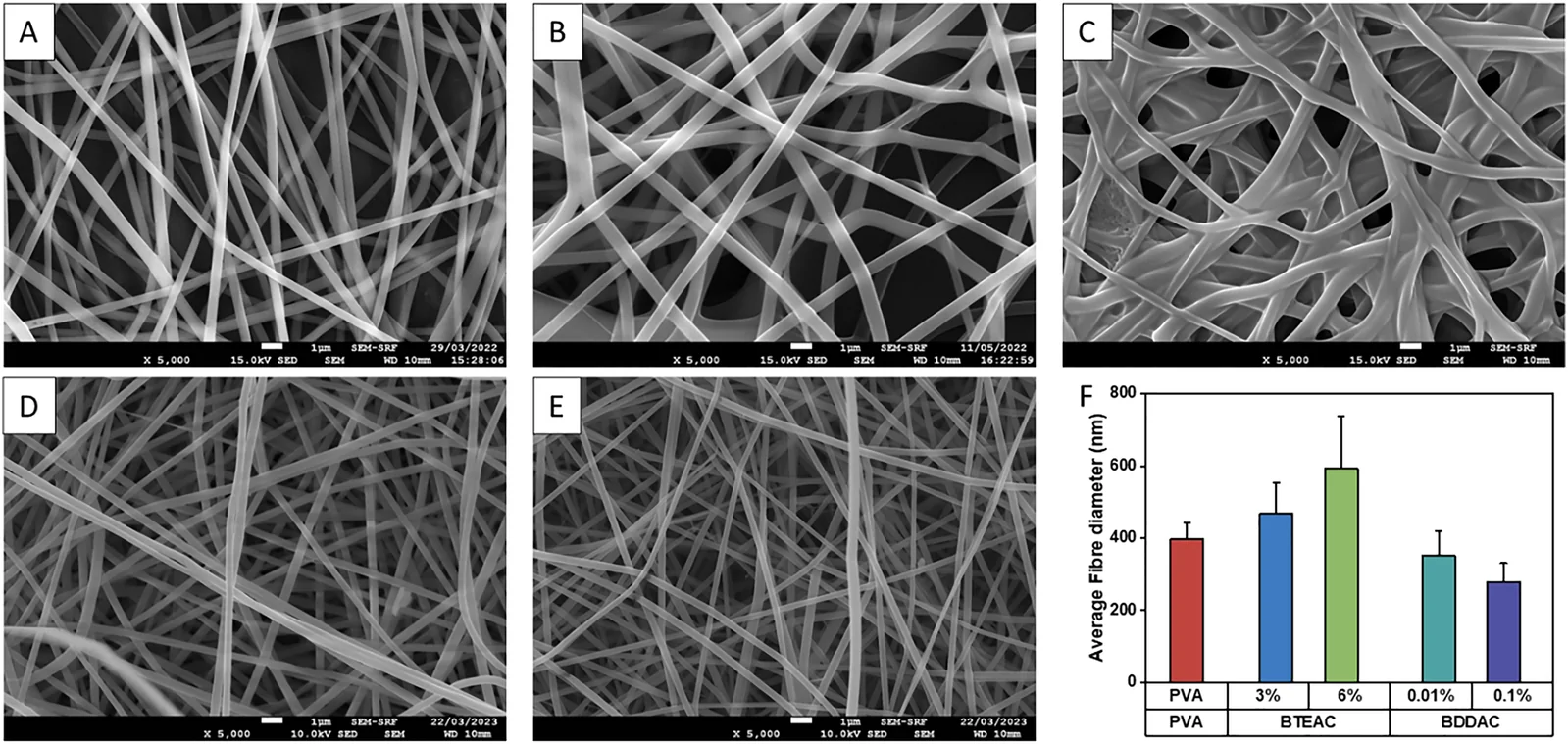
Representative SEM pictures of the electrospun (A) 9% PVA, (B) 9% PVA-3% BTEAC, (C) 9% PVA-6% BTEAC, (D) 9% PVA-0.01% BDMDAC and (E) 9% PVA-0.1% BDMDAC nanofibers produced by electrospinning, at 5000× magnification, scale bar is 1 µm, and (F) their average fiber diameter.

The reverse trend was observed when incorporating BDMDAC into the fibers; a decrease in BDMDAC concentration led to thicker fibers, measuring 279 ± 68 nm and 352 ± 51 nm at the concentration of 0.1% and 0.01%. However, the incorporation of BDMDAC slightly increased the viscosity, with solutions 0.01% and 0.1% BDMDAC having viscosities of 158 ± 8.6 and 190 ± 15 cP respectively. Additionally, conductivity slightly increased with BDMDAC content, reaching 1.81 ± 0.10 mS cm^−1^ for 0.01% addition and 2.4 ± 0.47 mS cm^−1^ for 0.1%. The surface tensions of these solutions were 42.3 ± 0.10 mN m^−1^ and 42 ± 1.0 mN m^−1^, respectively. The electrospinning stability and fiber yield were comparable between the two concentrations. However, considering the MIC and MBC results, which indicate effective antibacterial activity at concentrations on the µg mL^−1^ scale, the lower concentration of 0.01% BDMDAC was selected for subsequent studies.

The addition of 3% and 6% BTEAC to the PVA solutions resulted in an increase in fiber diameter. This can be attributed to the significant rise in conductivity, which introduced excess free charge. This led to intensified whipping behavior and reduced uniformity during spinning.^30,31^ Additionally, BTEAC affects interfacial tension and polymer chain entanglement, weakening chain cohesion and reducing the efficiency of elongation during flight.^32,33^ In the case of BDMDAC, the surface-active nature of BDMDAC reduced surface tension while increasing the solution's viscosity and conductivity. As a result, lower voltages could be used to produce bead-free fibers, leading to thinner-diameter fibers.^33–35^

### 3.3 Apparent density and porosity

An optimal porosity is crucial for promoting periodontal tissue regeneration and wound healing.^36,37^ The composition, theoretical density, apparent density and porosity of PVA nanofibers and QAC-loaded PVA nanofibers are presented in Table 2. Pure PVA nanofibers exhibited an apparent density of 0.04 ± 0.01 g cm^−3^ and an average porosity of 96.6%, whereas the PVA-BTEAC and PVA-BDMDAC nanofibers showed apparent densities of 0.36 ± 03 g cm^−1^ and 0.12 ± 0.001 g cm^−3^, with corresponding porosities of 64.3% and 90.4%, respectively. An inverse relationship between porosity and apparent density was observed, with higher QAC content in the fiber resulting in increased density and reduced porosity. This increase in density is primarily attributed to an increase in fiber crystallinity following the incorporation of QAC, which leads to a more compact structure, increased inter-fiber bonding, and a greater degree of void filling.^38–40^ Similar findings were reported by Abunahel *et al.* in 2018, for the apparent density of pure PVA nanofibers at approximately 0.03 g cm^−3^ using a density determination kit; upon the addition of metal nanoparticles, they observed an increase in density, a reduction in porosity and an enhancement in crystallinity.^40^

**Table 2:** Composition, theoretical density, apparent density, porosity and BET surface area of PVA, PVA-BTEAC and PVA-BDMDAC nanofibers

Sample	PVA wt%	BTEAC wt%	BDM-DAC wt%	Theoretical density (g cm^−3^)	Apparent density (g cm^−3^)	Porosity %	BET surface area (m^2^ g^−1^)
PVA	9	0	0	1.25^41^	0.04 ± 0.01	96.7 ± 0.4	21.2
PVA-BTEAC	9	3	0	1.01^42^	0.36 ± 0.03	65 ± 3.2	4.1
PVA-BDMDAC	9	0	0.01	1.25	0.12 ± 0.00	90.8 ± 0.3	18.7

### 3.4 Surface area and pore size distribution

The surface area and pore size distribution of PVA nanofibers and QAC-loaded nanofibers were determined using N_2_ adsorption/desorption, based on BET, HK, and BJH theories. The isotherms for both PVA and PVA-BDMDAC nanofibers resembled a type II adsorption isotherm, as shown in Fig. 2A, indicating the presence of macroporous structures. In contrast, the PVA-BTEAC nanofibers displayed a type I adsorption isotherm, which is characteristic of microporous structures, as classified by the International Union of Pure and Applied Chemistry (IUPAC).^43^ As shown in Table 2, the BET surface areas for the PVA, PVA-BTEAC, and PVA-BDMDAC nanofibers were measured at 21.2 m^2^ g^−1^, 4.6 m^2^ g^−1^, and 18.7 m^2^ g^−1^, respectively. Their average pore sizes were 3.3 nm, 2.7 nm, and 2.8 nm, respectively. The pore size distribution results from the HK and BJH models (Fig. S6) confirmed the presence of micropores, mesopores, and macropores within these nanofibers. The inclusion of BTEAC and BDMDAC led to a reduction in the fiber surface area due to the densification of the fiber structure, which increased crystallinity, thereby decreasing both porosity and surface area.^33^ Additionally, the PVA-BTEAC nanofibers exhibited a thicker fiber structure, resulting in a significant decrease in pore size and specific surface area per unit mass.^44^

**Fig. 2:**
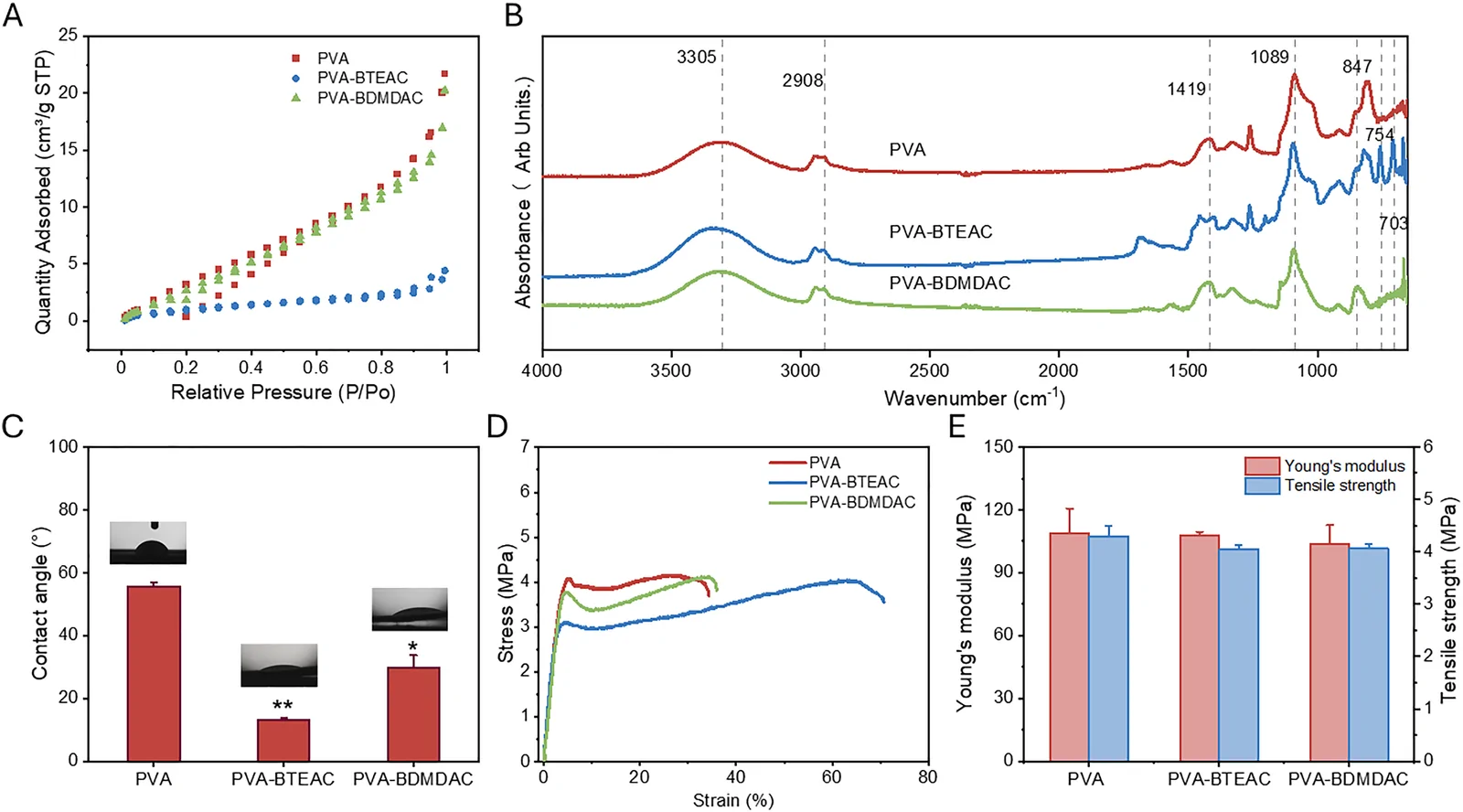
Characterization of PVA, PVA-BTEAC and PVA-BDMDAC nanofibers: (A) nitrogen adsorption/desorption isothermal plot, (B) FTIR spectra, (C) water contact angle, (D) stress–strain curves and their (E) mechanical properties.

### 3.5 Chemical characterization: FTIR

The chemical confirmation of optimized electrospun PVA and QAC-loaded PVA nanofibers were determined *via* FTIR, as shown in Fig. 2B. The PVA nanofibers have a wide absorption peak at 3305 cm^−1^, corresponding to O–H stretching. The absorption peaks at 2908, 1419, 1089 and 847 cm^−1^ are ascribed to C–H asymmetric stretching, CH_2_ bending, C–O stretching and C–C stretching, respectively.^17,45^ The PVA-BTEAC nanofibers have peaks at 754 cm^−1^ and 703 cm^−1^ that are attributed to the stretching vibration of the C–H bond in the benzyl group of the BTEAC molecule, and a peak at 703 cm^−1^ that corresponds to the stretching vibration of the C–Cl bond.^46^ As BDMDAC is a long alkyl chain QAC and is similar in chemical composition to PVA, no observable differences between the FTIR of the pure PVA nanofibers and the PVA-BDMDAC nanofibers are observed.

### 3.6 Wettability: contact angle

The wettability of electrospun PVA and QAC-loaded PVA nanofibers was evaluated by measuring their contact angles, as shown in Fig. 2C. Pure PVA nanofibers exhibited an average contact angle of 55.6 ± 1.4°, reflecting their hydrophilic nature due to the abundance of –OH groups, as previously reported.^26^ Upon incorporation of BTEAC and BDMDAC, a significant reduction in the contact angle was observed, with values of 13.3 ± 0.6° and 29.9 ± 3.9°, respectively, which indicates enhanced wettability. This decrease can be attributed to the presence of hydrophilic quaternary ammonium groups that increase the fiber's affinity for water.^47^ Moreover, a higher loading of BTEAC relative to BDMDAC results in a greater abundance of hydrophilic groups, leading to a lower contact angle. Similar findings were reported by Park *et al.*, who prepared quaternary ammonium-functionalized polyvinylidene fluoride/graphene oxide fibers subsequently coated with PVA; these fibers also exhibited enhanced hydrophilicity due to the combined presence of PVA and QAC.^48^

### 3.7 Mechanical properties

Nanofibers intended for dental applications must exhibit appropriate mechanical integrity and sufficient flexibility to withstand deformation during placement and intraoral use without fracture. Accordingly, the mechanical properties of electrospun PVA and QAC-loaded PVA nanofibers were investigated, with their respective stress–strain curves and mechanical properties presented in Fig. 2D and E. The incorporation of QACs into the nanofibers modestly reduced Young's modulus and tensile strength whilst considerably enhancing failure strain, indicating a shift from a stiffer towards a more ductile mechanical response. PVA nanofibers exhibited the highest Young's modulus of 109.0 ± 11.6 MPa, which decreased upon the addition of BTEAC and BDMDAC to 107.5 ± 2.0 MPa and 103.8 ± 8.9 MPa, respectively. The tensile strengths of the PVA-BTEAC and PVA-BDMDAC nanofibers (4.0 ± 0.1 MPa and 4.1 ± 0.1 MPa) were lower compared to pure PVA nanofibers (4.3 ± 0.2 MPa). However, the introduction of QAC significantly enhanced fiber ductility, as indicated by the increased failure strains for PVA-BTEAC (76.0 ± 4.0%) and PVA-BDMDAC (51.7 ± 2.1%) nanofibers compared with pure PVA nanofibers. This behavior can be attributed to the effect of QAC incorporation on increased fibrous crystallinity and reduced porosity, thus lowering stiffness and enhancing flexibility.^35^ Similar findings have been reported previously.^25^ Under dry test conditions, the QAC-loaded nanofibers maintained adequate integrity for specimen handling and demonstrated a greater failure strain compared to unmodified PVA. These results suggest a promising initial evaluation of the mechanical behavior of the dry electrospun mats. PVA can absorb water, which may lead to hydration-induced plasticization. Additionally, the stiffness and structural integrity of PVA may change after it is placed in PBS or artificial saliva. Future wet-state tensile testing, conducted after equilibration in artificial saliva or simulated gingival crevicular fluid at 37 °C, along with studies on handling, retention, and cyclic loading, will further clarify the mechanical performance of these materials in conditions relevant to periodontal-pocket applications.

### 3.8 Thermal stability

Thermal stability is a critical parameter for nanofiber-based drug delivery systems, as it influences processing, storage, and long-term performance under physiological and sterilization conditions. TGA and DSC were employed to evaluate the thermal stability of electrospun PVA and QAC-loaded PVA nanofibers. The initial stage of TGA curve for PVA nanofibers was observed between 26–124 °C, as shown in Fig. 3A, corresponding to the evaporation of water molecules adsorbed on the fiber surface. Due to their enhanced hydrophilicity, PVA-BTEAC and PVA-BDMDAC nanofibers exhibited greater water loss in this stage (3.0% and 5.2%, respectively) compared to PVA (2.3%), alongside an extended temperature range. Both PVA and PVA-BDMDAC nanofibers exhibited a two-step degradation. Pure PVA nanofibers initially degraded between 124–338 °C, while the PVA-BDMDAC nanofibers initially degraded between 170–334 °C. This degradation corresponds to the first stage of PVA thermal degradation, involving dehydration and the loss of –OH groups, ultimately leading to chain scission.^26,49,50^ In contrast, the PVA-BTEAC nanofibers displayed a multi-step degradation process, with distinct stages occurring between 142–283 °C and 283–404 °C. The initial thermal decomposition of the PVA-BTEAC nanofibers is attributed to the degradation of the BTEAC compounds, resulting in an approximate 3% reduction in sample mass. Lee *et al.* found that in a poly(vinylidene fluoride-hexafluoropropylene (PVDF-HFP)/BTEAC mat, BTEAC began to decompose between 210 and 260 °C.^46^ Subsequently, the second degradation stage, observed between 338–574 °C, is attributed to the further degradation of by-products and the formation of carbonized residues.^49^

**Fig. 3:**
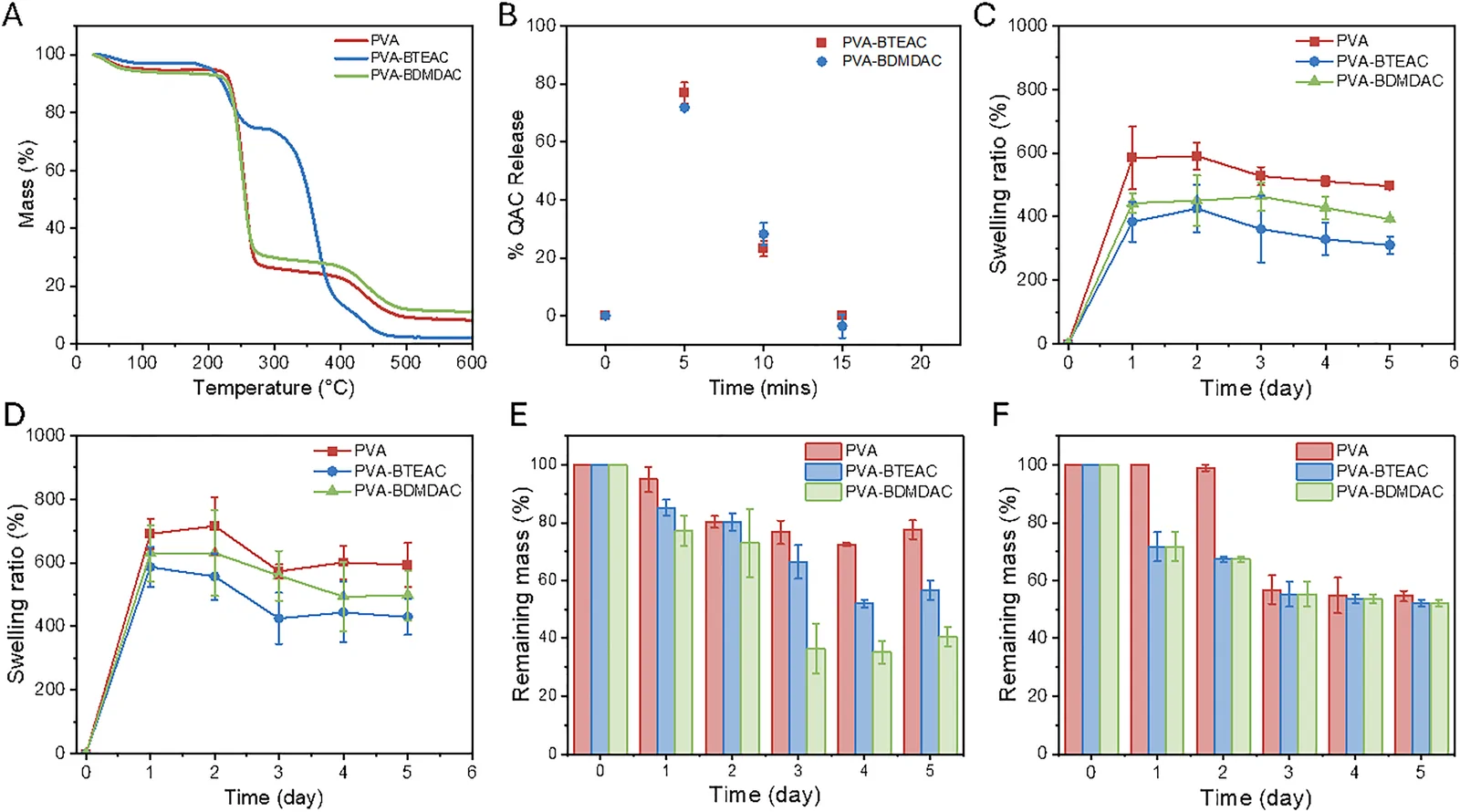
Characterization of PVA, PVA-BTEAC and PVA-BDMDAC nanofibers: (A) TGA curves, (B) release profile of BTEAC and BDMDAC, swelling ratio in (C) PBS and (D) artificial saliva, and their % of weight remaining in (E) PBS and (F) artificial saliva.

The DSC curves of electrospun nanofibers produced from pure PVA, PVA-BTEAC, and PVA-BDMDAC are presented in Fig. S7. The PVA nanofibers exhibited a glass transition temperature (*T*_g_) at 100 °C. Upon the addition of BDMDAC to the PVA nanofibers, the peak maximum decreased to 95 °C. This can be attributed to the long alkyl chain structure of BDMDAC, which facilitates effective packing with the PVA polymer chains. In contrast, the incorporation of BTEAC increased the peak to 114 °C, attributing to the presence of additional hydrophilic groups that interact with the PVA polymer chains, thereby enhancing hydrogen bonding.^33^ Consequently, both BTEAC and BDMDAC introduce positive charges, generating strong intermolecular forces with the –OH groups of PVA. This improves its thermal stability, which increases the temperature where PVA degrades and is consistent with the findings of Park and Kim.^32^

### 3.9 *In vitro* drug release

Understanding the release behavior of antimicrobial agents from the nanofibers is essential for predicting therapeutic performance and ensuring effective local drug concentrations. The *in vitro* release profiles of PVA, PVA-BTEAC, and PVA-BDMDAC nanofibers were assessed by immersing the samples in deionized water for durations ranging from 5 to 20 minutes, as illustrated in Fig. 3B. The concentrations of BTEAC and BDMDAC released from the fibers were quantified using a standard calibration curve. Both PVA-BTEAC and PVA-BDMDAC nanofibers exhibited similar release behaviors, characterized by a rapid burst release within the first 5 minutes, corresponding to 76.8% and 71.8% of their total loadings, respectively. Complete release of encapsulated BTEAC and BDMDAC from the electrospun nanofibers into the aqueous medium occurred within 15 minutes. This rapid release can be attributed to two complementary factors: the highly hydrophilic nature of the PVA matrix, which promotes rapid fiber swelling and water penetration, and the intrinsic water solubility of BTEAC and BDMDAC, both of which favor immediate drug mobilization upon contact with aqueous media. The correlation between fiber wettability and release rate observed across the sample series is consistent with this interpretation, whereby more hydrophilic fibers exhibit faster initial drug mobilisation.^51^ Comparable behavior has been reported by Park and Kim, who observed a pronounced burst release from untreated electrospun BTEAC-PVA fibers at the first measurement time point,^32^ supporting the view that this kinetic profile is an intrinsic characteristic of unmodified PVA-QAC systems. Together, the inherently high surface area-to-volume ratio of the nanofibrous architecture further amplifies this effect,^52^ enabling rapid and complete release that generates an immediate and high local QAC concentration upon contact with the surrounding environment. This property has notable therapeutic implications, particularly in the context of antimicrobial applications. The current formulations are designed as fast-dissolving burst-release inserts intended for short-term local treatment following mechanical periodontal debridement, allowing for the rapid achievement of concentrations that exceed the bactericidal threshold and aiding in the disruption of residual biofilms. While complete release within 15 minutes may limit the duration of protection against bacterial recolonization, this quick-release behavior aligns well with the goal of immediate antimicrobial action following periodontal debridement.

For formulations achieving QAC concentrations above the corresponding bactericidal threshold, rapid release may promote prompt biofilm reduction while shortening the period during which bacteria are exposed to sub-inhibitory concentrations. This may represent a favourable feature for limiting opportunities for adaptive tolerance. Nevertheless, physiological dilution and heterogeneous penetration through the biofilm matrix may create local concentration gradients, potentially exposing bacteria in less accessible regions to sublethal QAC concentrations. Such exposure could promote adaptive responses, including increased efflux-pump activity, membrane modification, and biofilm-associated tolerance. Overall, the current findings support the potential of the nanofibre platform to provide rapid and effective local antimicrobial exposure following periodontal debridement, although repeated-exposure and resistance-development studies under clinically representative conditions are required to confirm this potential advantage. Future research will focus on optimizing the release profile by exploring techniques such as core–shell electrospinning to extend antimicrobial exposure when clinically beneficial.

### 3.10 Swelling and degradation behavior

Assessment of swelling behavior and degradation is essential for predicting structural stability and functional performance under physiological conditions. The PVA and QAC-loaded PVA nanofibers were soaked in PBS and artificial saliva over a period of five days for swelling ratio and degradation evaluation. Their swelling and degradation behavior are shown in Fig. 3C–F. In PBS, PVA nanofibers exhibited an initial weight loss of 5% with 584% swelling after day 1. And then, increasing degradation to a weight loss of 23% and slightly reduced swelling (497%) by day 5. In contrast, PVA-BTEAC nanofibers showed higher initial degradation (15%) and lower swelling (385%) after 1 day, reaching weight loss of 48% and further reduced swelling (330%) by day 4. Notably, PVA-BDMDAC nanofibers presented the highest initial degradation in PBS, with a 23% weight loss and 443% swelling after day 1, significantly increasing to 65% weight loss by day 4, accompanied by decreased swelling (428%). The quaternary ammonium groups from BTEAC and BDMDAC enhance ionic interaction with PBS, leading to weakening the hydrogen-bonded fibrous network and more weight loss after 5 days.^53^ As explained in the previous section, PVA-BDMDAC nanofibers have the most hydrophilic nature with higher porosity and lower crystallization than PVA-BTEAC nanofibers. Ionic interaction with phosphate salts is more effective, causing deeper structural breakdown and the highest mass loss.^25,33,54^

In artificial saliva, PVA nanofibers initially showed negligible degradation (0%) yet exhibited high swelling (691%) after day 1, subsequently experiencing notable degradation (45%) by day 3, with swelling stabilizing around 600%. The penetration of multivalent ions (Ca^2+^, Mg^2+^) into the fiber, disrupted hydrogen bonds, resulting in the increased degradation behaviour.^55^ PVA-BTEAC nanofibers displayed rapid initial degradation of 28% with 587% swelling after 1 day, increasing to 45% degradation with a corresponding decrease in swelling (426%) by day 3. Similarly, PVA-BDMDAC nanofibers initially showed lower degradation (6%) and significant swelling (630%) after 1 day, followed by substantial degradation of 48% with reduced swelling (560%) by day 3. The positively charged ions in QACs caused rapid ionic exchange, leading to initial degradation in QAC-loaded nanofibers.^53,56^

Overall, the swelling behavior of these samples in PBS and artificial saliva is similar. This could be due to the differences in the porosity of the fibers. PVA nanofibers have the highest porosity and therefore a higher swelling capacity, whilst the PVA-BTEAC nanofibers have a lower porosity, with a more compact structure, which would not have the capacity to uptake as much solution and would therefore have the lowest swelling rate.^57^ Similar findings were reported by Pisani *et al.*, who assessed the swelling and degradation behavior of electrospun poly-l-lactide-*co*-poly-*ε*-caprolactone (PLA-PCL) scaffolds in artificial saliva, and found that with higher porosity of the sample, more weight loss was seen.^58^ In this study, QAC-loaded nanofibers have a higher swelling rate and weight remaining in artificial saliva compared to the swelling and degradation behavior of the samples in PBS.

### 3.11 Antibacterial analysis

#### 3.11.1 Antimicrobial activity on planktonic bacteria

Planktonic bacteria represent a distinct physiological state from biofilm-associated cells, with important implications for antimicrobial susceptibility.^59^ Therefore, the antimicrobial activity of electrospun PVA-BTEAC and PVA-BDMDAC nanofibers was evaluated against planktonic *S. aureus*, *E. coli*, *S. mutans*, and *P. gingivalis*. Pure PVA supported growth comparable to the growth control (GC), confirming no intrinsic antimicrobial activity.

Against *S. aureus* (as shown in Fig. 4A and B), PVA-BTEAC demonstrated a transient effect after 4 hours, with approximately 1 to 2 log reductions at concentrations of 10 and 15 mg mL^−1^. However, by 24 hours, regrowth eliminated any significant difference compared to the GC. In contrast, PVA-BDMDAC was markedly more potent. At 4 hours, a concentration of 0.001 mg mL^−1^ resulted in a 1.7 log reduction, and concentrations of 0.010 mg mL^−1^ or higher yielded no recoverable colonies. After 24 hours, concentrations of 0.005 mg mL^−1^ and above achieved complete killing.

**Fig. 4:**
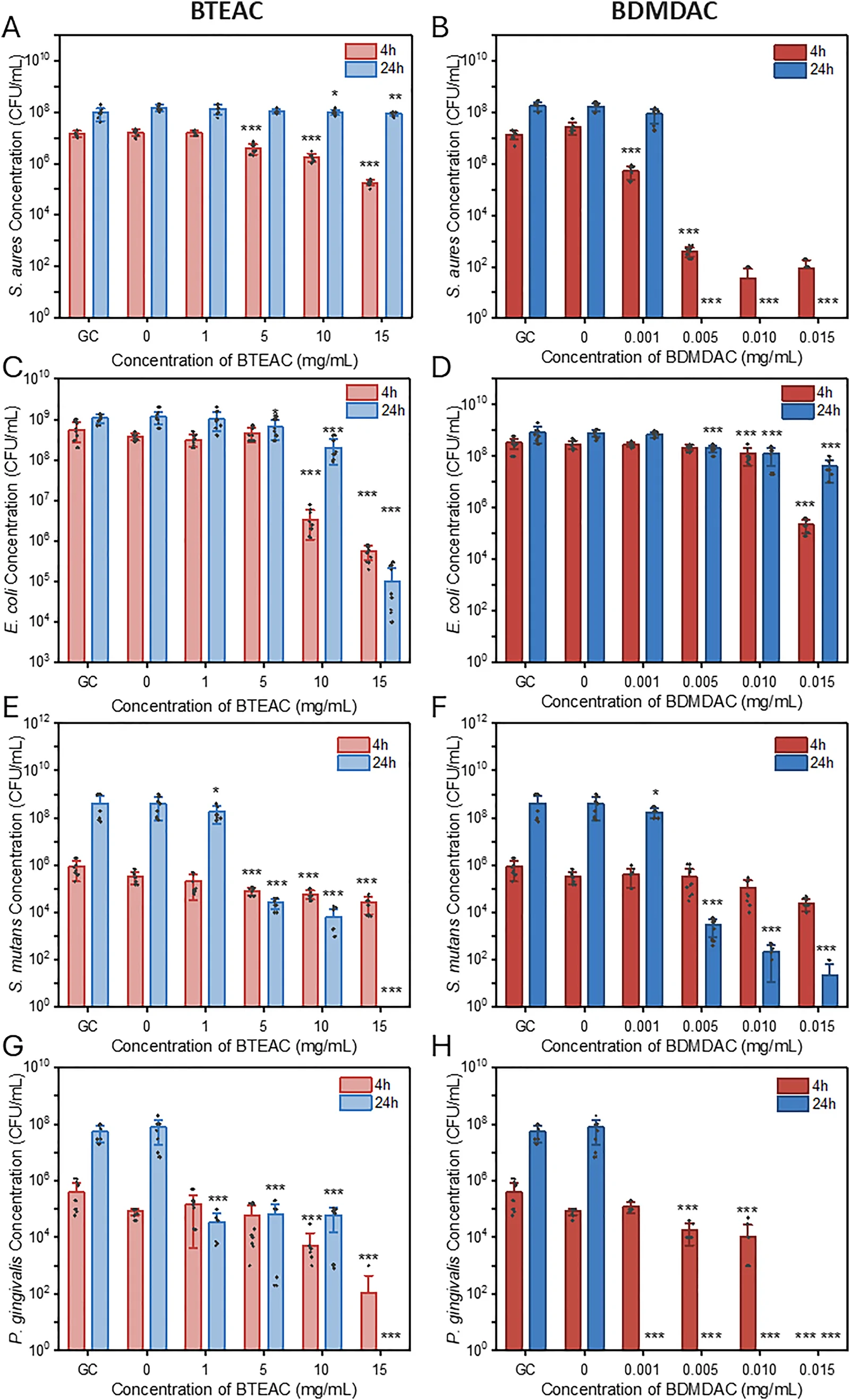
The antimicrobial activity of electrospun nanofibers against planktonic bacteria after 4 h and 24 h incubation: (A) PVA-BTEAC and (B) PVA-BDMDAC nanofibers against *S. aureus*; (C) PVA-BTEAC and (D) PVA-BDMDAC nanofibers against *E. coli*; (E) PVA-BTEAC and (F) PVA-BDMDAC nanofibers against *S. mutans*; (G) PVA-BTEAC and (H) PVA-BDMDAC nanofibers against *P. gingivalis*. *p < 0.05, **p < 0.01, and ***p < 0.001 from the corresponding concentrations with growth control.

For *E. coli*, PVA-BTEAC produced reductions of 2.0 and 2.8 log at 4 hours with concentrations of 10 and 15 mg mL^−1^, respectively, as presented in Fig. 4C and D. However, at 24 hours, efficacy at 10 mg mL^−1^ declined to approximately 0.8 log, while the 15 mg mL^−1^ concentration increased to a 4 log reduction. PVA-BDMDAC exhibited concentration-dependent activity: at 4 hours, concentrations of 0.005 mg mL^−1^ had a limited effect, 0.010 mg mL^−1^ resulted in a 0.4 log reduction, and 0.015 mg mL^−1^ achieved a 3.1 log reduction. After 24 hours, concentrations of 0.005 and 0.010 mg mL^−1^ achieved 0.6 and 0.8 log reductions, respectively. In contrast, the 0.015 mg mL^−1^ group showed partial regrowth, leading to a 1.3 log reduction, indicating reduced sustained activity at the highest concentration.

To mimic oral oxygen gradients, *S. mutans* were tested under both aerobic and anaerobic conditions. Under aerobic conditions (Fig. 4E and F), PVA-BTEAC achieved reductions of 1.0, 1.2, and 1.5 log at 4 hours for concentrations of 5, 10, and 15 mg mL^−1^, respectively. After 24 hours, 4.2 and 4.8 log reduction was observed at higher concentrations, with no recoverable colonies at 15 mg mL^−1^. Under anaerobic conditions (Fig. S7), activity was lower, with reductions of 0.5, 0.6, and 0.8 log at 4 hours across the same concentrations, increasing to 1 log at 15 mg mL^−1^ by 24 hours. PVA-BDMDAC, however, was highly effective against planktonic *S. mutans*: at 24 hours, concentrations of 0.005, 0.010, and 0.015 mg mL^−1^ produced reductions of 5.1, 6.3, and 7.3 log, respectively. Under anaerobic conditions, while activity decreased, it remained significant, with reductions greater than 2 log at 4 hours across 0.005 to 0.015 mg mL^−1^, and 2.9, 4.6, and 5.2 log reductions at 24 hours for the respective concentrations. For *P. gingivalis* (Fig. 4G and H), PVA-BTEAC displayed activity at 10 and 15 mg mL^−1^, resulting in reductions of 1.2 and 2.9 log at 4 hours. By 24 hours, all tested concentrations achieved reductions of greater than 3 log, with complete eradication at the highest concentration. PVA-BDMDAC was even more effective; at 4 hours, a concentration of 0.001 mg mL^−1^ was comparable to the control, while concentrations above 0.001 mg mL^−1^ resulted in 0.7 to 0.9 log reductions. Concentrations of 0.005 to 0.015 mg mL^−1^ achieved complete killing by 4 hours, and all tested concentrations resulted in no recoverable colonies after 24 hours.

The observed differences in antimicrobial efficacy between BTEAC and BDMDAC are primarily attributed to their amphiphilic structures. The positively charged quaternary ammonium groups in both compounds interact electrostatically with the negatively charged bacterial cell wall components, including peptidoglycan and lipopolysaccharide, displacing divalent cations and disrupting membrane integrity.^60,61^ BTEAC demonstrates greater binding affinity for divalent cations in Gram-negative bacteria, as discussed in Section 3.10, thereby contributing to its higher efficacy against these pathogens. BDMDAC, by contrast, preferentially inserts into the phospholipid bilayer, destabilizing membranes and ultimately leading to bacterial cell death, which accounts for its enhanced efficacy against Gram-positive bacteria.^62–64^ In the case of *S. mutans*, aerobic conditions may trigger the accumulation of intracellular reactive oxygen species, synergizing with QAC-induced membrane disruption and collectively increasing bacterial susceptibility beyond that attributable to electrostatic interaction alone.^65,66^ These compound- and species-specific differences in membrane-targeting behavior provide the mechanistic basis for the broad-spectrum planktonic efficacy observed across the bacterial panel, and underpin the antibiofilm activity discussed in the following section.

#### 3.11.2 Biofilm inhibition

Biofilm-associated bacteria exhibit altered physiology and enhanced tolerance to antimicrobial agents compared to their planktonic counterparts, presenting a major challenge for periodontal therapy. As a result, the antibiofilm activity of the electrospun PVA-BTEAC and PVA-BDMDAC nanofibers was evaluated in terms of biofilm inhibition, and biofilm morphology was examined by SEM. For *S. aureus*, PVA-BTEAC nanofibers demonstrated no measurable antimicrobial activity at 24 hours, as shown in Fig. 5A and B. No reduction in viable counts was observed compared to the control group, and extensive bacterial attachment and mature biofilm structures were clearly visible on the fiber surface. In contrast, PVA-BDMDAC nanofibers significantly suppressed *S. aureus* biofilm formation. At a concentration of 0.001 mg mL^−1^ BDMDAC, a 0.8 log reduction in bacterial viability was observed, and noticeable decrease in the number of adherent cells was observed by SEM. Increasing the BDMDAC concentration beyond 0.001 mg mL^−1^ further decreased biofilm formation, resulting in log reductions of 4.5, 4.3, and 5.1, along with smoother, mostly cell-free fiber surfaces shown in the SEM images (Fig. S7).

**Fig. 5:**
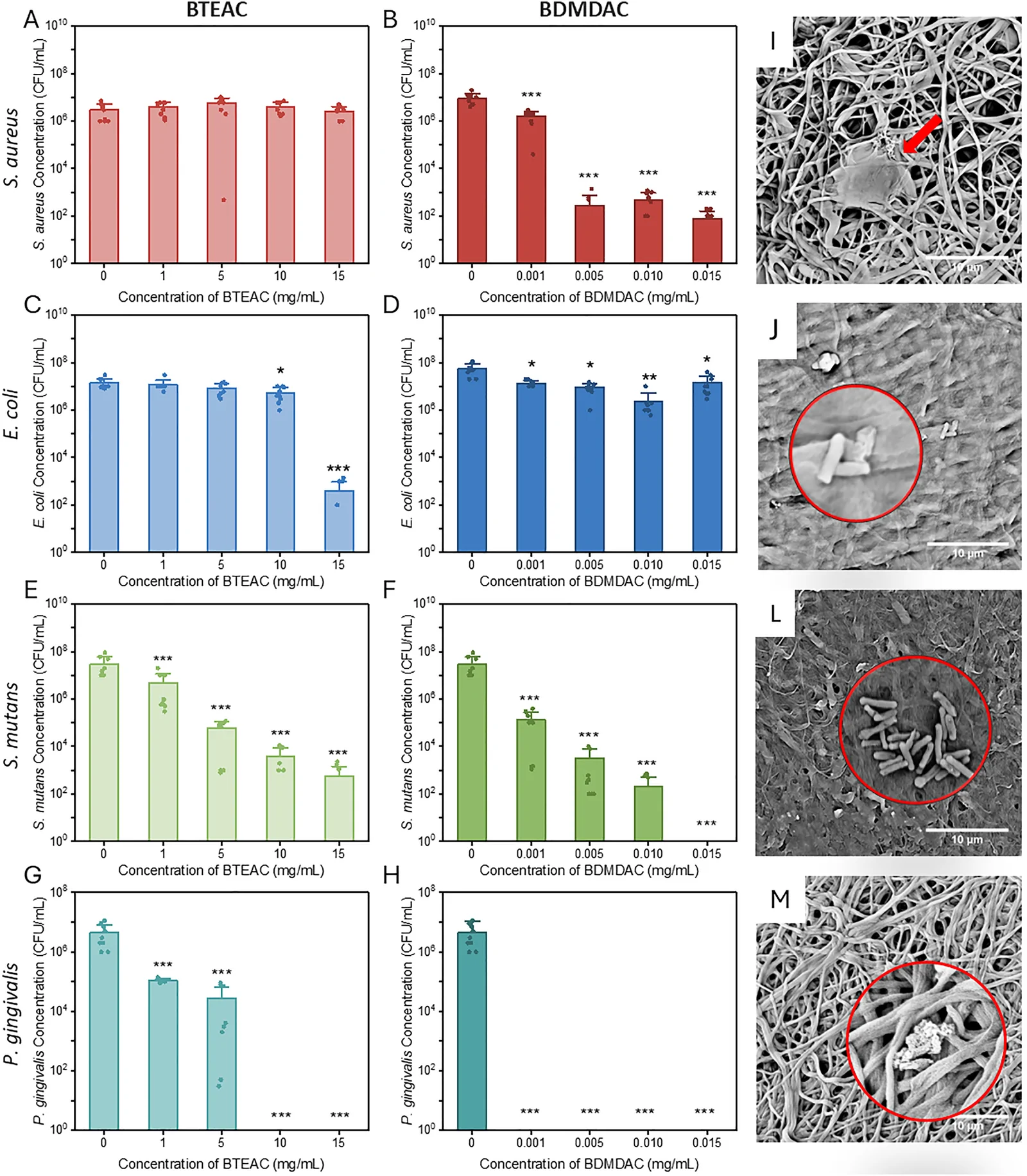
The biofilm inhibition of electrospun nanofibers against bacteria after 24 h incubation. Quantified biofilm inhibition of (A) PVA-BTEAC and (B) PVA-BDMDAC nanofibers against *S. aureus*; (C) PVA-BTEAC and (D) PVA-BDMDAC nanofibers against *E. coli*; (E) PVA-BTEAC and (F) PVA-BDMDAC nanofibers against *S. mutans*; (G) PVA-BTEAC and (H) PVA-BDMDAC nanofibers against *P. gingivalis*. And the representative images of cell damage: (I) 15 mg mL^−1^ of BTEAC and (J) 0.015 mg mL^−1^ of BDMDAC against *E. coli*; (L) 5 mg mL^−1^ of BTEAC and (M) 0.015 mg mL^−1^ of BDMDAC against *P. gingivalis.* *p < 0.05, **p < 0.01, and ***p < 0.001 from the corresponding concentrations with control nanofibers.

In the case of *E. coli*, the control exhibited a dense and architecturally heterogeneous biofilm that appeared as irregular “bumps,” obscuring the underlying fibers, as shown in Fig. S8. On PVA-BTEAC nanofibers (Fig. 5C and D), a 4.6 log reduction was observed at a concentration of 15 mg mL^−1^; however, at lower concentrations, the inhibition was negligible. This corresponded to the SEM images, where a thin, patchy biofilm and notable morphological abnormalities were observed at 15 mg mL^−1^, including the rounding of typically rod-shaped cells and signs of membrane damage, with leaked intracellular contents and debris (indicated by red arrows in Fig. 5I). The PVA-BDMDAC nanofibers demonstrated modest but consistent inhibition of *E. coli* across all tested concentrations, achieving at least a 0.6 log reduction. The maximum activity was noted at 0.010 mg mL^−1^ (1.4 log reduction), which declined to approximately 0.6 log at 0.015 mg mL^−1^. SEM images (Fig. 5J) revealed increasingly wrinkled cell surfaces and irregular envelopes, suggesting altered membrane permeability and structural damage.^67,68^

For *S. mutans* (Fig. 5E and F), PVA-BTEAC nanofibers inhibited biofilm formation under aerobic conditions in a concentration-dependent manner. At a concentration of 1 mg mL^−1^, biofilm viability decreased by >0.8 log, with further increases in BTEAC loading resulting in reductions of up to 4.7 log. SEM images of the control showed characteristic chains of coccus-shaped *S. mutans* forming compact aggregates, as shown in Fig. S9, while BTEAC-loaded nanofibers exhibited progressively sparser coverage at higher concentrations. PVA-BDMDAC nanofibers demonstrated even more vigorous activity under aerobic conditions. At a concentration of 0.001 mg mL^−1^ BDMDAC, a 2.4 log reduction in biofilm viability was observed, and no detectable viable biofilm remained at 0.015 mg mL^−1^. SEM micrographs showed residual clusters at 0.001 mg mL^−1^, transitioning to smoother, largely cell-free fiber surfaces with scattered debris at higher concentrations, indicating membrane damage. Under anaerobic conditions (Fig. S10), PVA-BTEAC nanofibers showed limited activity after 24 hours, with no significant reduction in *S. mutans* biofilm. In contrast, although the effectiveness of PVA-BDMDAC nanofibers decreased compared to aerobic conditions, they still exhibited strong antibiofilm performance, resulting in 3.5 log and 4.6 log reductions at concentrations of 0.010 and 0.015 mg mL^−1^, respectively.

When tested against *P. gingivalis* (Fig. 5G and H), PVA-BTEAC nanofibers produced reductions of 1.6 log and 2.2 log at concentrations of 1 mg mL^−1^ and 5 mg mL^−1^, respectively, with complete inhibition observed at concentrations greater than 5 mg mL^−1^. SEM images of the control group (Fig. S11) showed extensive colonization with dense biofilm clusters. As BTEAC concentrations increased, surface coverage decreased, and at 5 mg mL^−1^, clear shrinkage and indentations of *P. gingivalis* cells were evident (Fig. 5L). In contrast, all samples of PVA-BDMDAC nanofibers achieved complete inhibition of *P. gingivalis* biofilm formation after 24 hours. SEM images displayed clean, well-defined fibers with minimal to no intact cells; as demonstrated in Fig. 5M, at 0.015 mg mL^−1^, only remnants consistent with lysed bacteria were occasionally observed.

Building on the membrane-disruption mechanisms established above, the antibiofilm activity of QACs operates through additional concentration-dependent pathways. At bactericidal concentrations, membrane disruption prevents stable surface attachment and early cell aggregation,^69,70^ whilst at sub-inhibitory concentrations, QACs suppress extracellular polymeric substance synthesis and progressively reduce sessile cell viability^69,71^ Species-specific membrane characteristics further modulate these responses: the high surface negative charge density of *E. coli* accelerates ion exchange and membrane disruption,^72,73^ whilst the loosely packed membrane architecture of *P. gingivalis* renders it particularly susceptible to cationic disruption.^60,61,74^ The broad-spectrum efficacy observed across both planktonic and biofilm assays is consistent with the burst-release kinetics characterized above, whereby immediate and complete QAC mobilization generates supralethal concentrations capable of overcoming the diffusion barrier of the extracellular polymeric matrix, representing a therapeutic objective distinct from the prevention of early attachment associated with sustained sub-inhibitory delivery.

### 3.12 Cell viability assay

To evaluate the cytocompatibility of electrospun PVA-BTEAC and PVA-BDMDAC nanofibers, a 4 hours and 24 hours leachate MTT assay was conducted in accordance with ISO 10993 guidelines.^29,75^ The results are presented in Fig. 6. The PVA nanofibers were observed to be non-cytotoxic. When various concentrations of BTEAC were incorporated into the fibers, over 80% cell viability was maintained at the 4 h time point. Although reductions in cell viability were observed at 10 mg mL^−1^ and 15 mg mL^−1^ of BTEAC, with 92.7% and 93.4% cell viability, respectively, these remained within the non-cytotoxic range. After 24 h, the 15 mg mL^−1^ BTEAC sample was the only formulation found to be cytotoxic, with 53.4% cell viability observed. In comparison, all concentrations of the tested PVA-BDMDAC nanofibers were non-cytotoxic, with cell viability exceeding 90% across all groups. The lowest cell viability for L929 fibroblasts was observed in the 0.015 mg mL^−1^ BDMDAC group after 4 hours of incubation, at 87.3%. However, cell viability increased to 95.9% after 24 hours. Notably, the 0.005 mg mL^−1^ BDMDAC group seemed to promote cell proliferation and metabolic activity, as evidenced by a cell viability value exceeding 131%.

**Fig. 6:**
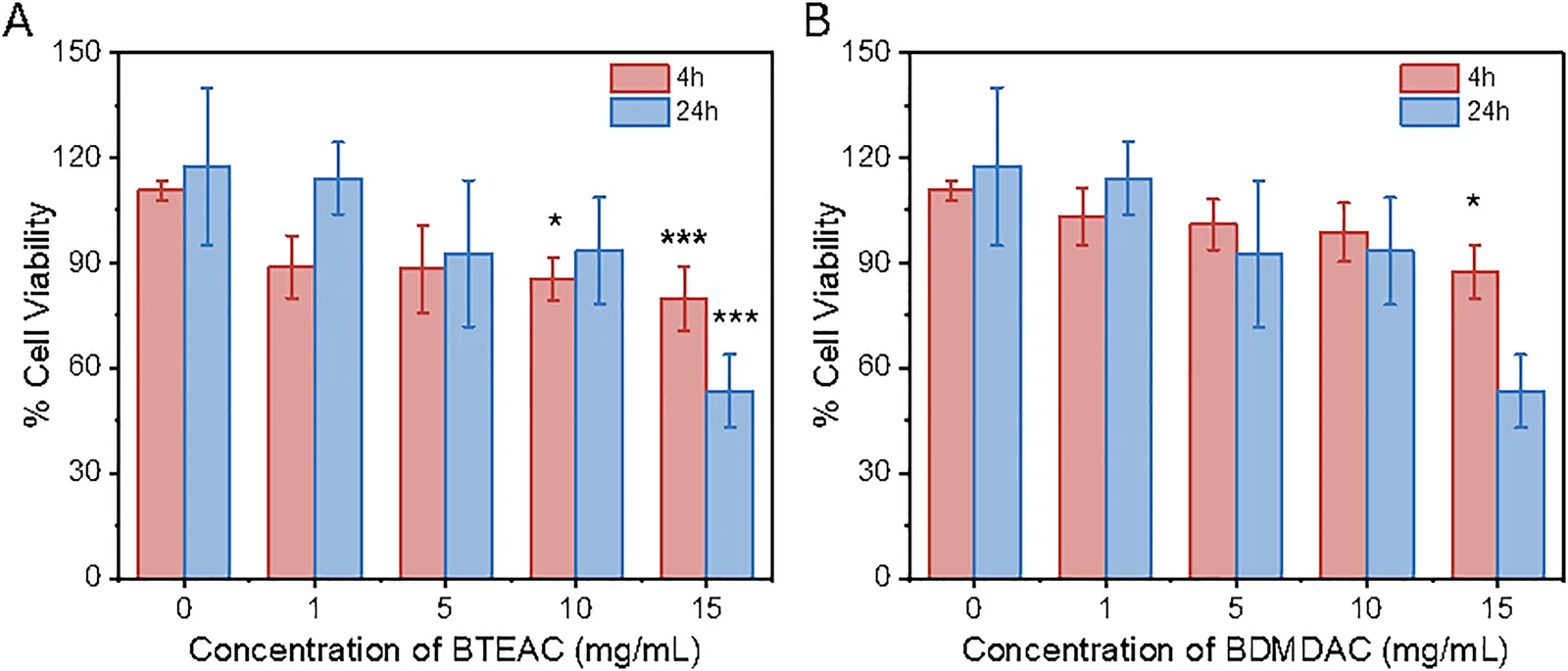
Cytotoxicity of electrospun (A) PVA-BTEAC and (B) PVA-BDMDAC nanofibers on L929 fibroblasts after 4 and 24 hours. *p < 0.05, **p < 0.01 and ***p < 0.001 from the corresponding concentrations with control nanofibers.

QACs typically exhibit preferential toxicity towards bacteria over mammalian cells. This difference is due to variations in membrane composition and charge, which affect how cationic surfactants interact with these cells.^76,77^ In line with this, leachates from PVA-BDMDAC were well tolerated by L929 fibroblasts across the tested concentration range. In contrast, high doses of PVA-BTEAC exhibited time-dependent cytotoxicity. This cytotoxicity is likely due to an acute burst release of the compound from the fibers (Section 3.9), resulting in temporarily elevated local concentrations that reduce cell viability. Similar burst-driven effects on L929 cells have been reported for other QAC systems.^76,78^ The >131% viability at 0.005 mg mL^−1^ BDMDAC aligns with a hermetic response, where a low dose of a stressor offers a beneficial or stimulatory effect, whereas a higher dose becomes toxic or inhibitory. This phenomenon has been observed with certain cationic antimicrobials.^79^

## 4. Conclusion

This study develops broad-spectrum antimicrobial QAC-releasing electrospun nanofibers for the localized treatment of periodontal infections. Incorporation of QACs increased fiber density and crystallinity, leading to reduced porosity and surface area, while enhanced intermolecular interactions conferred improved thermal stability. The resulting nanofibers exhibited high wettability, measurable dry-state tensile integrity, and sustained structural stability upon immersion in artificial saliva, supporting their physicochemical suitability for application at the oral mucosal interface.

Both PVA-BTEAC and PVA-BDMDAC formulations demonstrated rapid *in vitro* drug release and broad-spectrum antimicrobial activity against clinically relevant planktonic and biofilm-associated pathogens. Importantly, antimicrobial efficacy was strongly dependent on QAC structure, concentration, and environmental conditions. PVA-BTEAC nanofibers exhibited selective antimicrobial efficacy against Gram-negative and dental pathogens, including *E. coli*, *S. mutans*, and *P. gingivalis*. In contrast, PVA-BDMDAC showed strong bactericidal effects against Gram-positive pathogens such as *S. aureus*, *S. mutans*, and *P. gingivalis*. Most QAC concentrations were found to be non-cytotoxic, with only the highest BTEAC dosage showing cytotoxic effects due to burst release.

These results highlight the potential of molecular design and nanofiber engineering to tailor physicochemical properties, release behaviour, and antimicrobial performance within a single platform. The rapid and complete release of QACs generates an immediate supralethal concentration at the application site, a kinetic profile well suited to disrupting established periodontal biofilms. This burst-release behaviour may also reduce the risk of antimicrobial tolerance by rapidly exceeding the bactericidal threshold and limiting sub-inhibitory exposure. However, dilution by gingival crevicular fluid, adsorption to proteins and tissues, and uneven diffusion through the biofilm may create local concentration gradients that expose some bacteria to sublethal QAC levels. Future studies should therefore evaluate release under physiologically relevant flow conditions, mucosal biocompatibility, and antibiofilm efficacy against clinically relevant multispecies periodontal communities, together with repeated-exposure and resistance-development assays to determine whether the system can provide sufficiently rapid and uniform antimicrobial exposure.

Overall, this work demonstrates that optimized QAC-loaded PVA nanofibers combine well-characterized physicochemical properties with potent, broad-spectrum antimicrobial activity, positioning them as promising candidates for localized periodontal therapy. BDMDAC-loaded nanofibers show a strong potential for deployment within the dynamic and microbiologically complex environment of periodontal pockets, offering a material-driven strategy to address persistent oral biofilm infections.

## Conflicts of interest

There are no conflicts to declare.

## Supplementary Material

MA-007-D6MA00633G-s001

## Data Availability

The data that support the findings are available on request from the first author (Ting Chen: t.chen42@liverpool.ac.uk) and corresponding author (Raechelle D'Sa: r.dsa@liverpool.ac.uk). The supplementary information provides additional details on the optimisation of electrospun PVA nanofibers. See DOI: https://doi.org/10.1039/d6ma00633g.
