# Supplementary material for: Electrospun poly(vinyl alcohol) nanofibers loaded with quaternary ammonium compounds for localized eradication of periodontal biofilms
Source: Mater Adv. 2026 Sep 3;7(19):9710–26. doi: 10.1039/d6ma00633g (PMC13637459; doi:10.1039/d6ma00633g)
Supplement: Supplementary file 1 [file MA-007-D6MA00633G-s001.pdf]

Supplementary Information (SI) for Materials Advances.
This journal is © The Royal Society of Chemistry 2026

Supplementary Information

Electrospun Poly(vinyl alcohol) Nanofibers Incorporating Quaternary Ammonium Compounds for Localized Eradication of Periodontal Biofilms

Ting Chen [a], Kelly Nicola [a], Pedro Leonidas Oseliero Filho[b], Megan Carr[b], Alexander Ciupa[b], Jenny Aveyard [a], Raechelle A. D'Sa*[a]

[a] *School of Engineering, University of Liverpool, Brownlow Hill, Liverpool, L69 3GH, UK*

[b] *Materials Innovation Factory, University of Liverpool, Oxford Street, Liverpool, L7 3NY, UK*

Table S1. Solution properties and fiber diameter of electrospun solutions.

Solution	Viscosity (cP)	Surface Tension (mN/m)	Conductivity (mS/cm)	Fibre Diameter (nm)
8% PVA	71.5 ± 1.3	-	-	387.1 ± 49.1
9% PVA	136.3 ± 7.8	52.1 ± 1.5	1.7 ± 0.1	390.6 ± 60.1
10% PVA	177.5 ± 4.3	-	-	635.5 ± 97.1
9% PVA - 3% BTEAC	192.7 ± 7.9	51.8 ± 0.7	8.5 ± 0.3	469.2 ± 83.2
9% PVA - 6% BTEAC	197.2 ± 2.1	46.9 ± 0.3	14.2 ± 0.4	592.2 ± 145.1
9% PVA – 0.01% BDMDAC	158.2 ± 8.6	42.3 ± 0.1	1.81 ± 0.1	351.9 ± 51.0
9% PVA – 0.1% BDMDAC	190.0 ± 15.2	41.6 ± 1.0	2.39 ± 0.47	278.5 ± 67.9

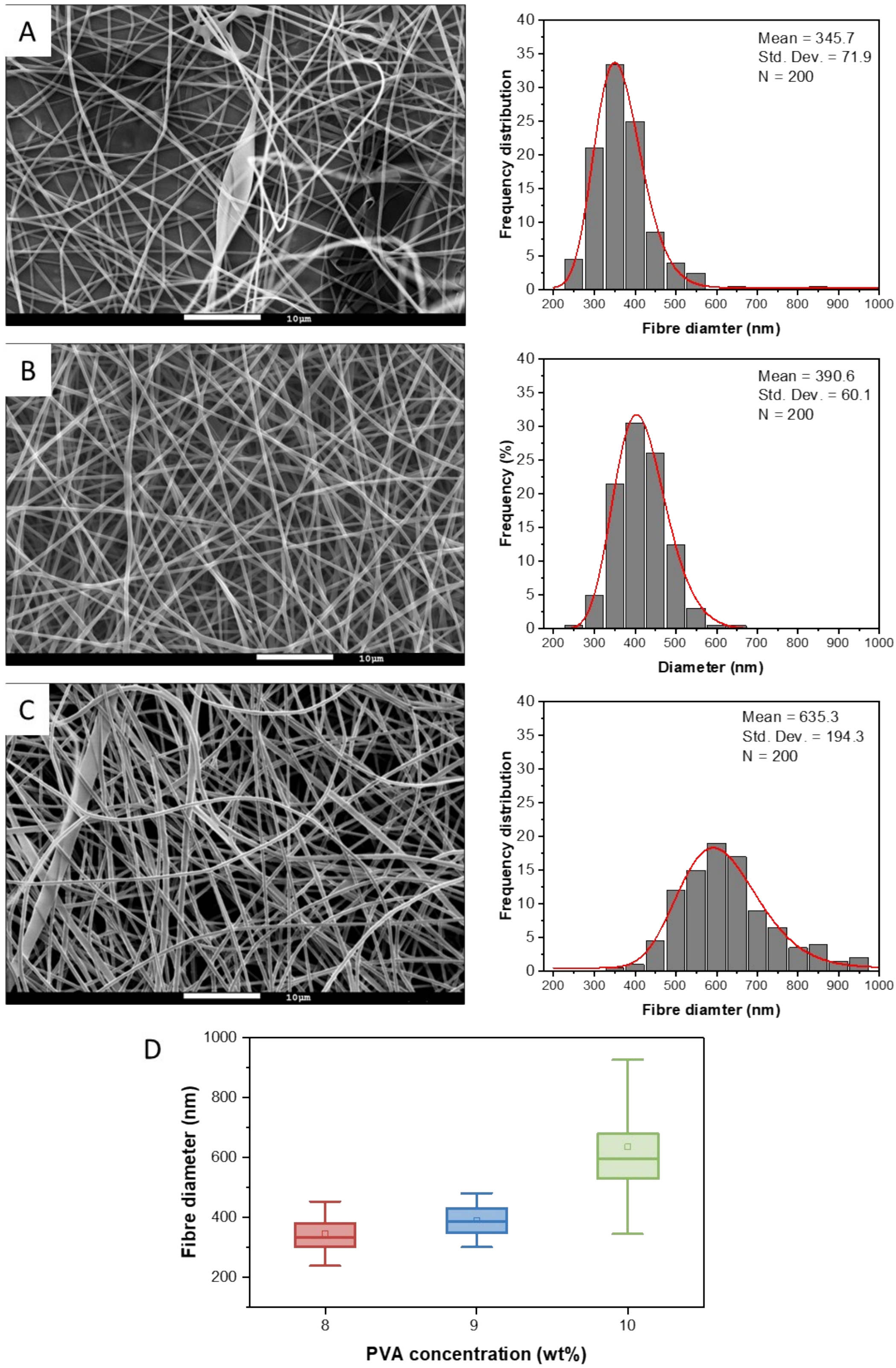

Figure S1. SEM images of electrospun PVA nanofibres at concentrations of (A) 8 wt%, (B) 9 wt%, (C) 10 wt%, along with their corresponding fibre diameter distributions and (D) average diameter.

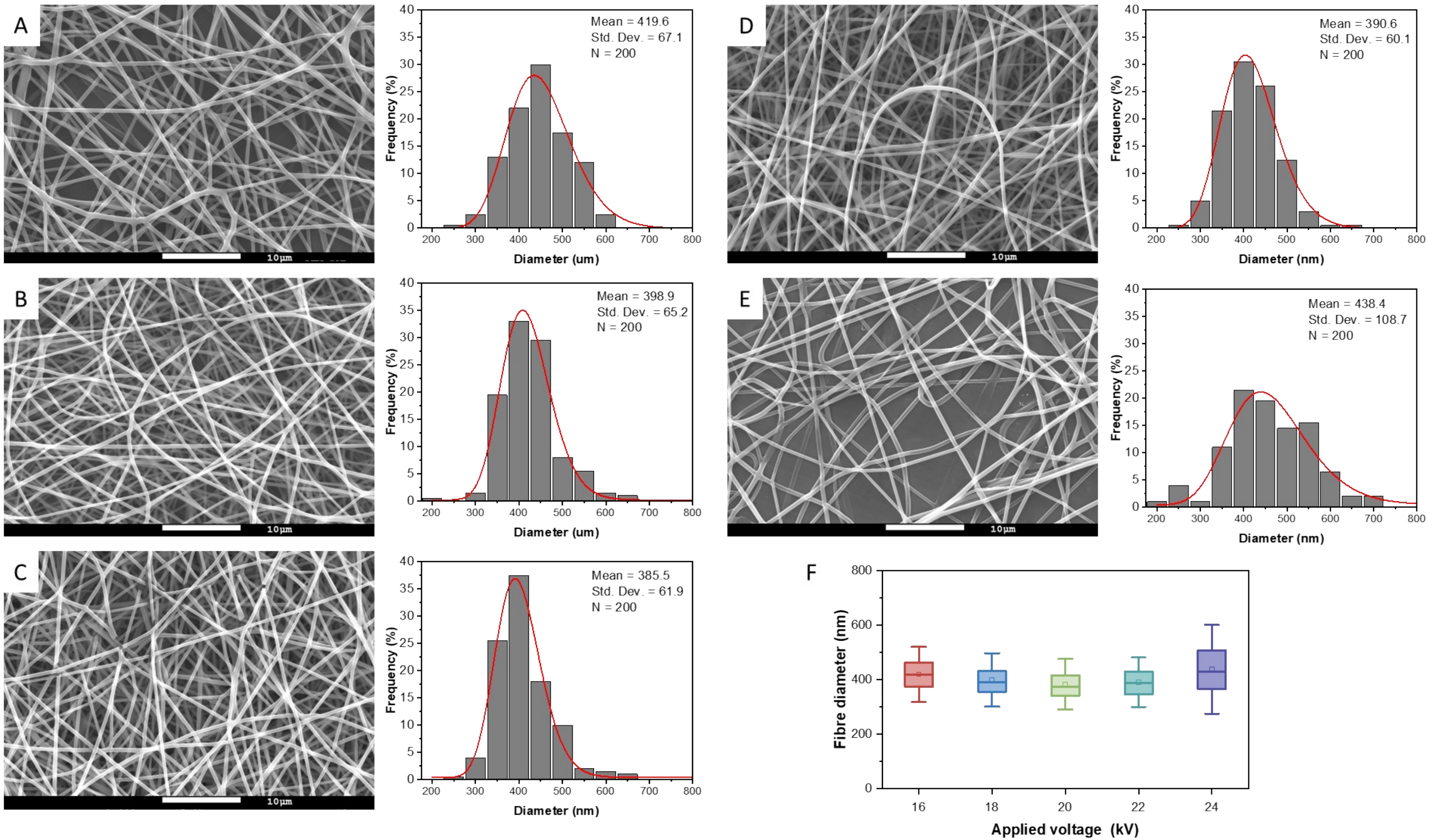

Figure S2. SEM images of electrospun PVA nanofibres fabricated at different processing voltages. (A) 16 kV, (B) 18 kV, (C) 20 kV, (D) 22 kV, (E) 24 kV, along with their corresponding fibre diameter distributions, and (F) average diameters.

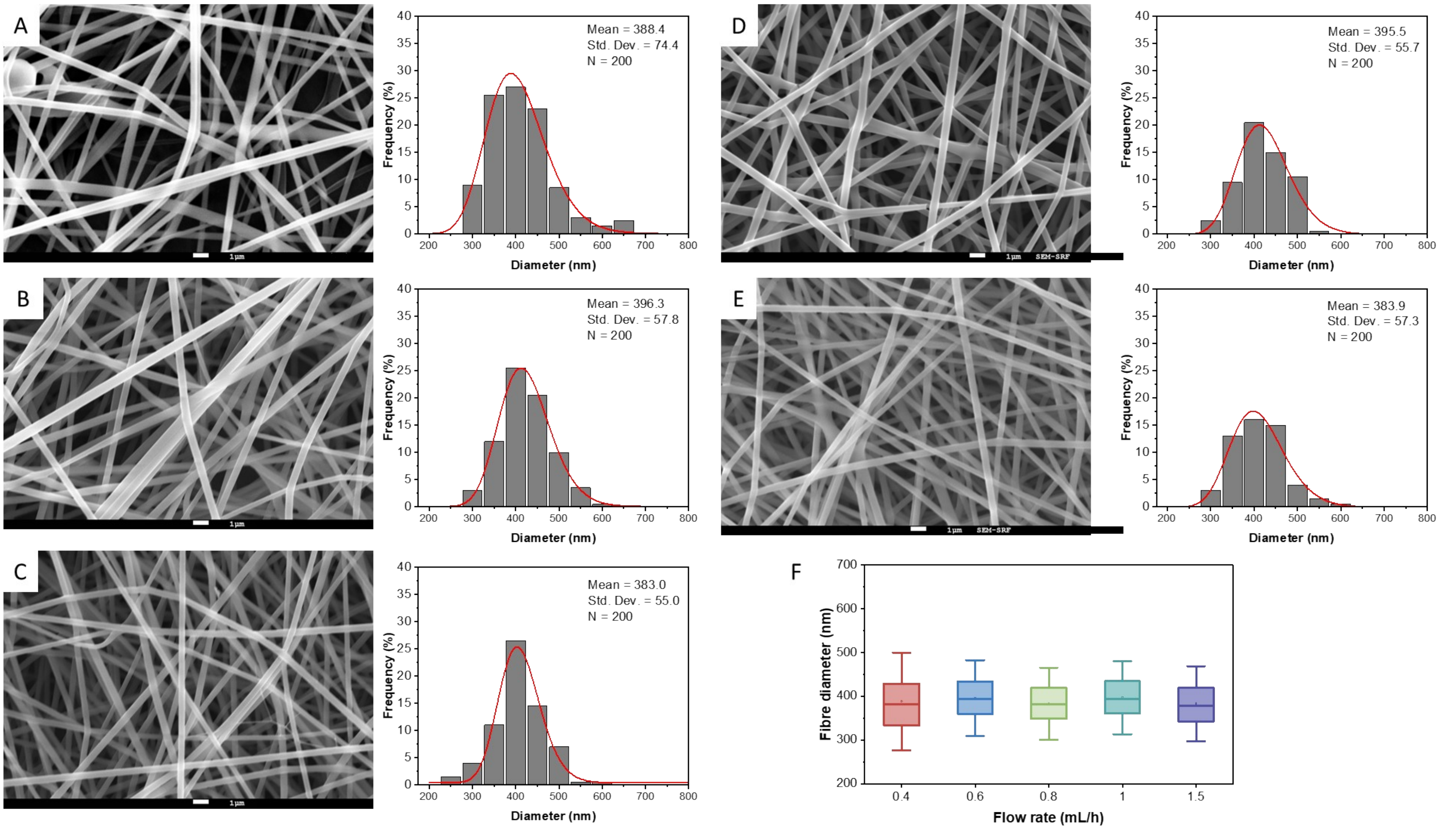

Figure S3. SEM images of electrospun PVA nanofibres fabricated at different flow rate. (A) 0.4 mL/h, (B) 0.6 mL/h, (C) 0.8 mL/h, (D) 1.0 mL/h, (E) 1.5 mL/h, along with their corresponding fibre diameter distributions and (F) average diameter.

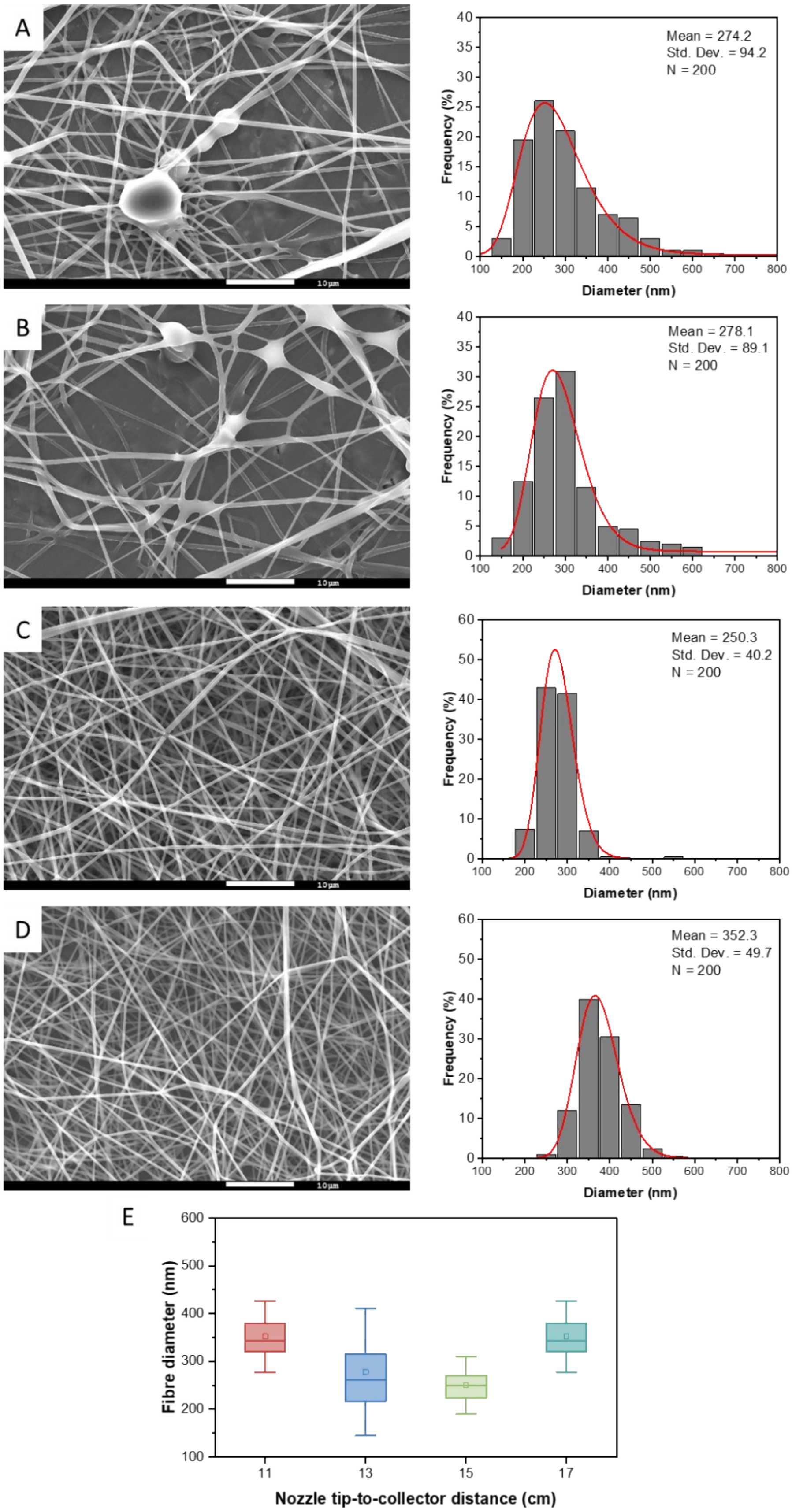

Figure S4. SEM images of electrospun PVA nanofibres fabricated at different flow rate. (A)11 cm, (B) 13 cm, (C) 15 cm, (D) 17 cm, along with their corresponding fibre diameter distributions and (E) average diameter.

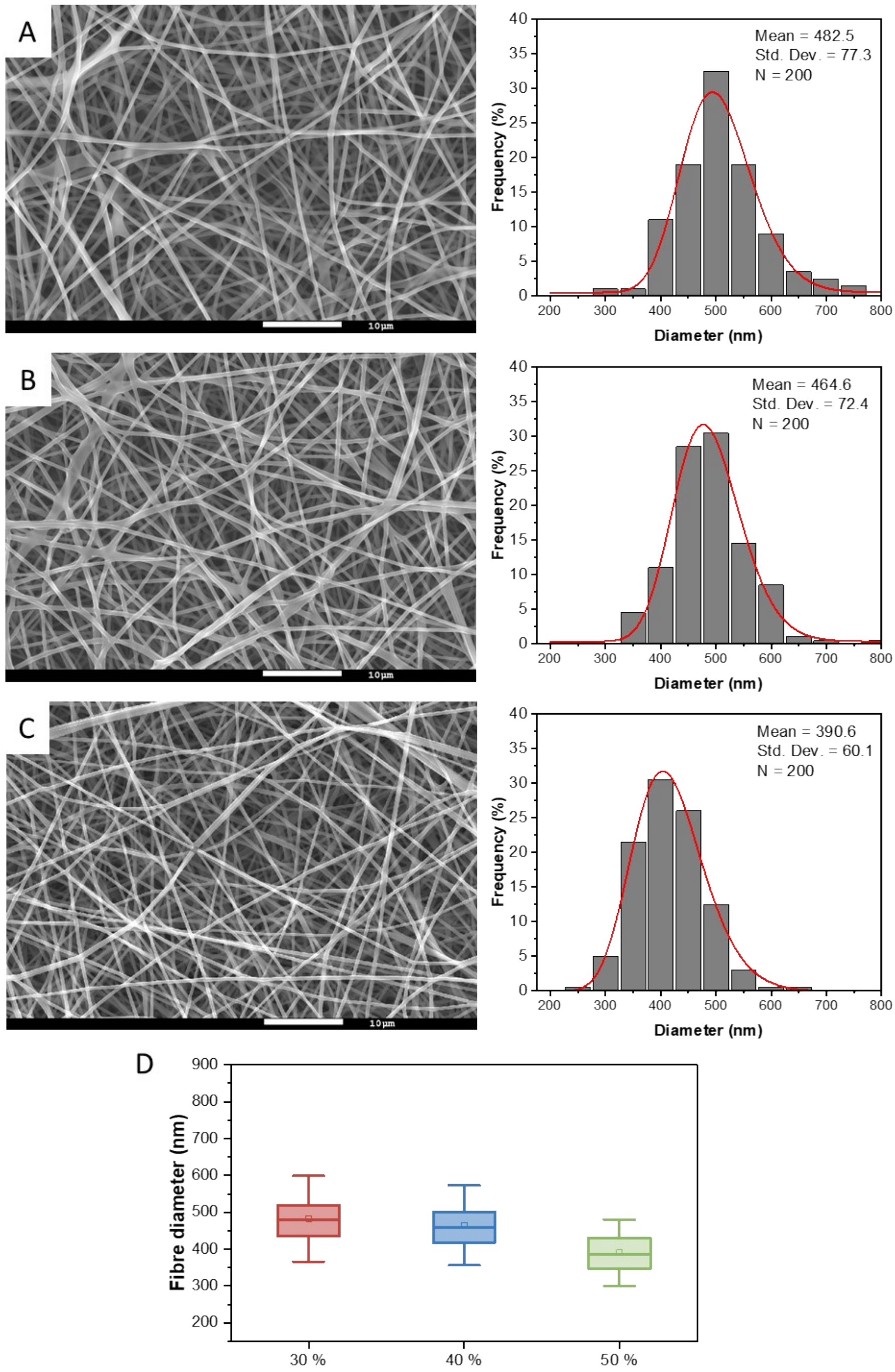

Figure S5. SEM images of electrospun PVA nanofibres fabricated at different RH. (A) 30% RH, (B) 40% RH, (C) 50% RH, along with their corresponding fibre diameter distributions and (D) average diameter.

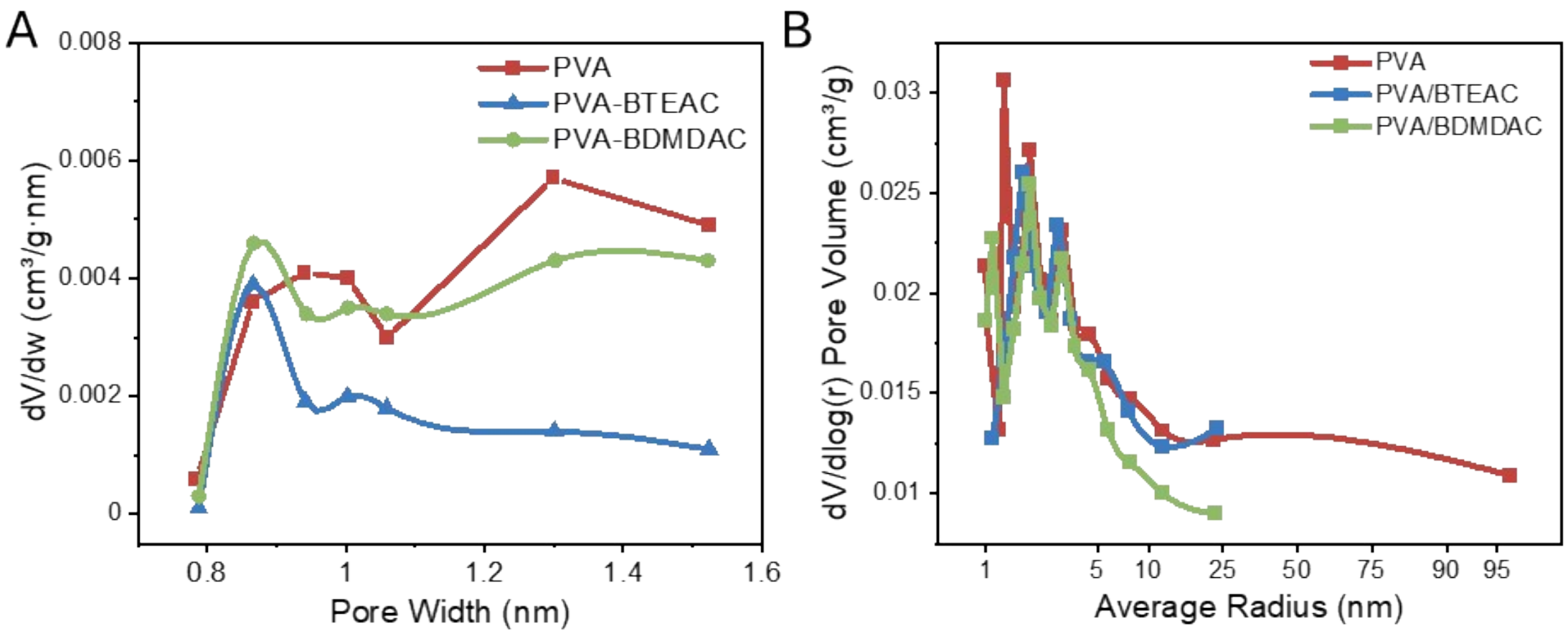

Figure S6. Pore size distribution of electrospun nanofibres: (A) HK and (B) BJH distribution of PVA, PVA-BTEAC and PVA-BDMDAC nanofibres.

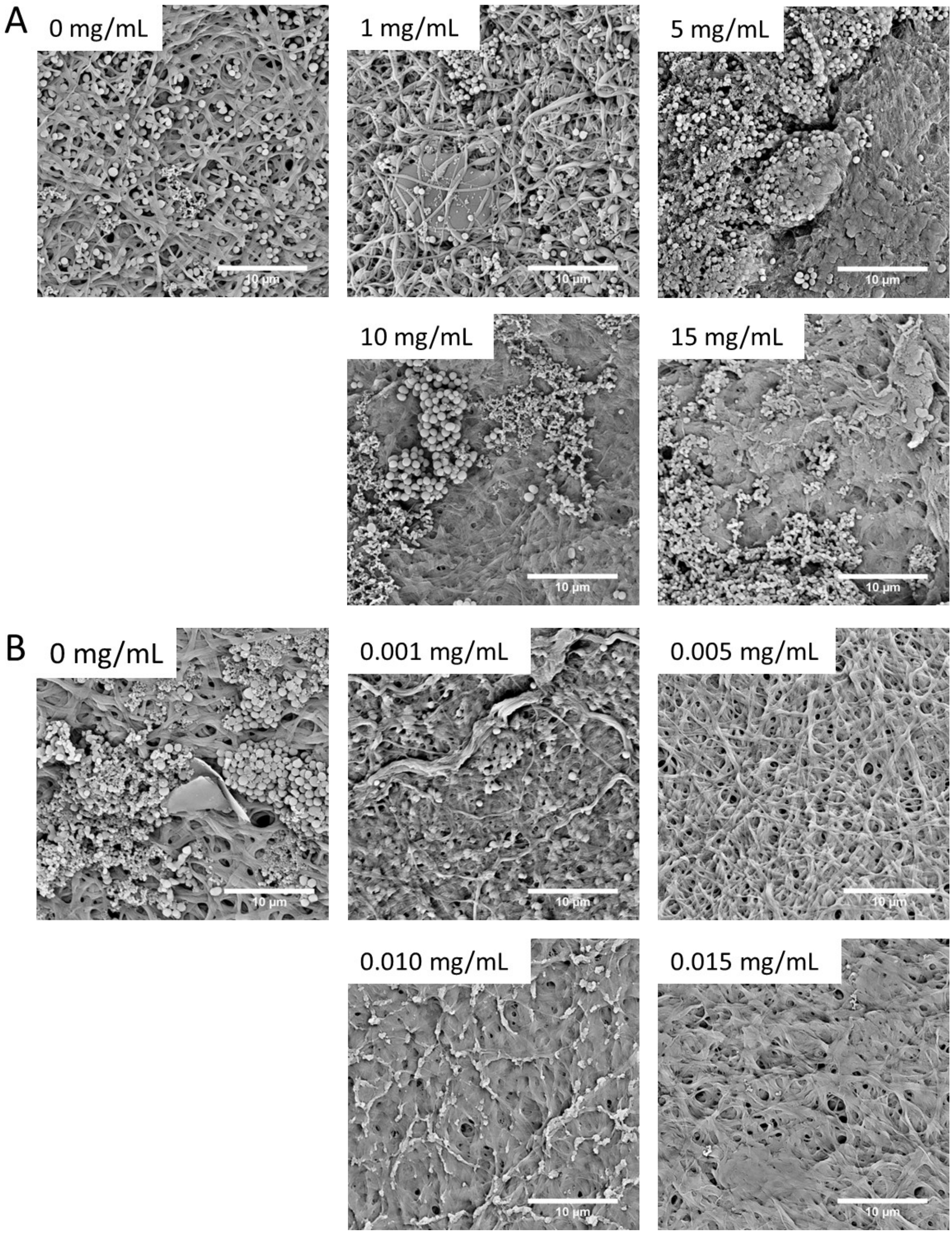

Figure S7. SEM images of 24 h *S. aureus* biofilm exposed to electrospun nanofibres. (A) PVA-BTEAC nanofibres surface at concentrations from 0 – 15 mg/mL; and (B) PVA-BDMDAC nanofibres surface at concentrations from 0 – 0.015 mg/mL. Scale bars = 10 µm.

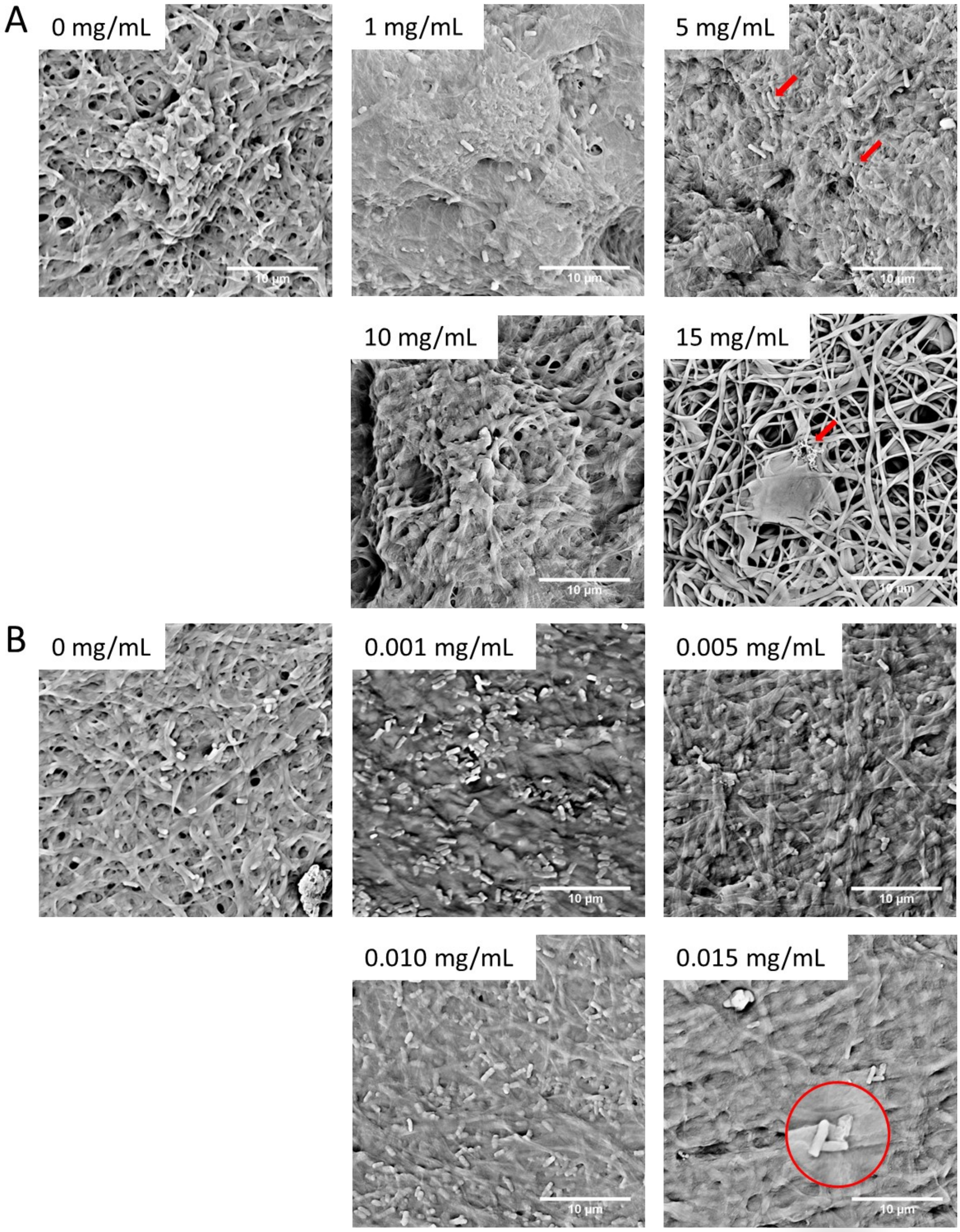

Figure S8. SEM images of 24 h *E. coli* biofilm exposed to electrospun nanofibres. (A) PVA-BTEAC nanofibres surface at concentrations from 0 – 15 mg/mL; and (B) PVA-BDMDAC nanofibres surface at concentrations from 0 – 0.015 mg/mL. Scale bars = 10 µm.

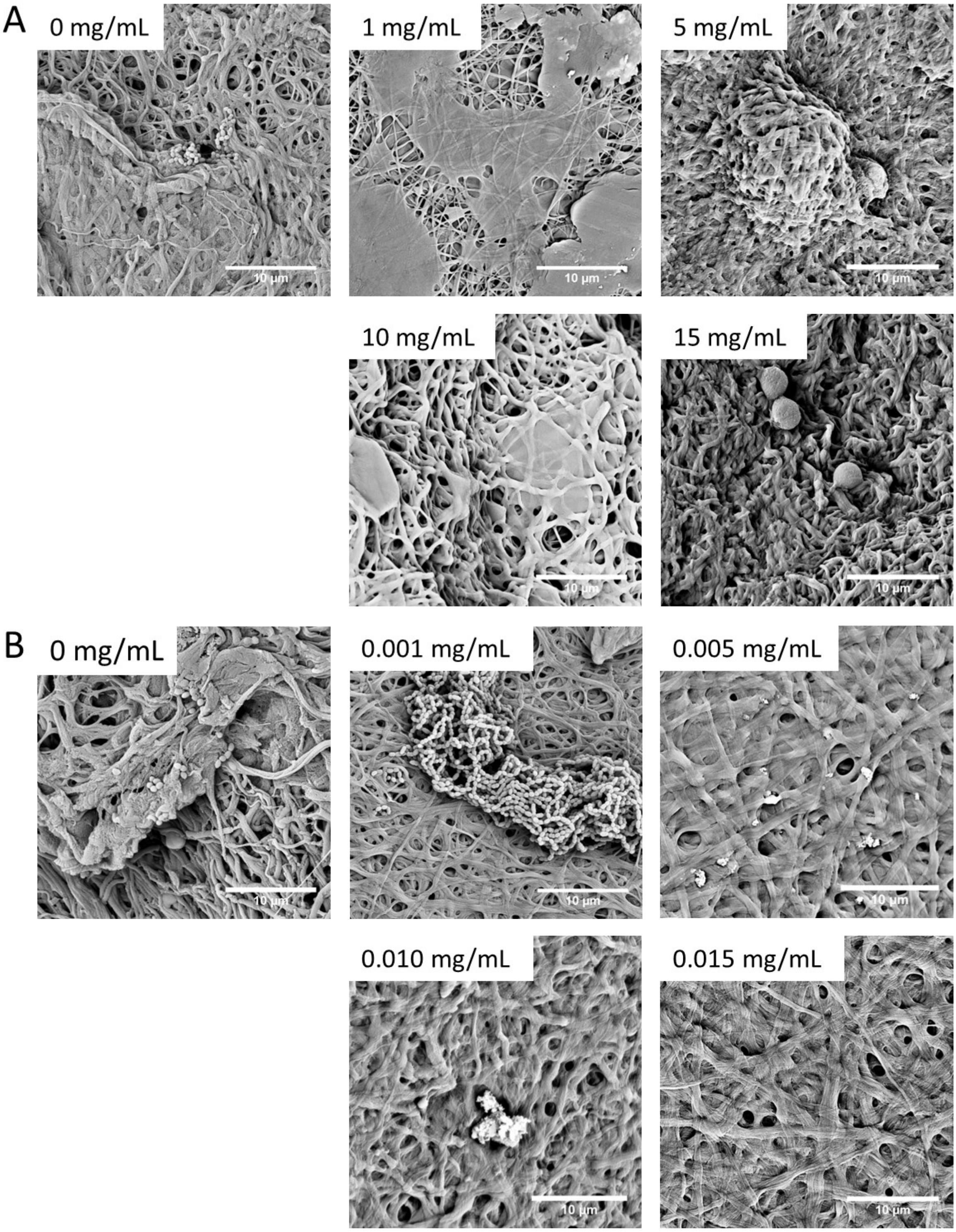

Figure S9. SEM images of 24 h *S. mutans* biofilm exposed to electrospun nanofibres. (A) PVA-BTEAC nanofibres surface at concentrations from 0 – 15 mg/mL; and (B) PVA-BDMDAC nanofibres surface at concentrations from 0 – 0.015 mg/mL. Scale bars = 10 µm.

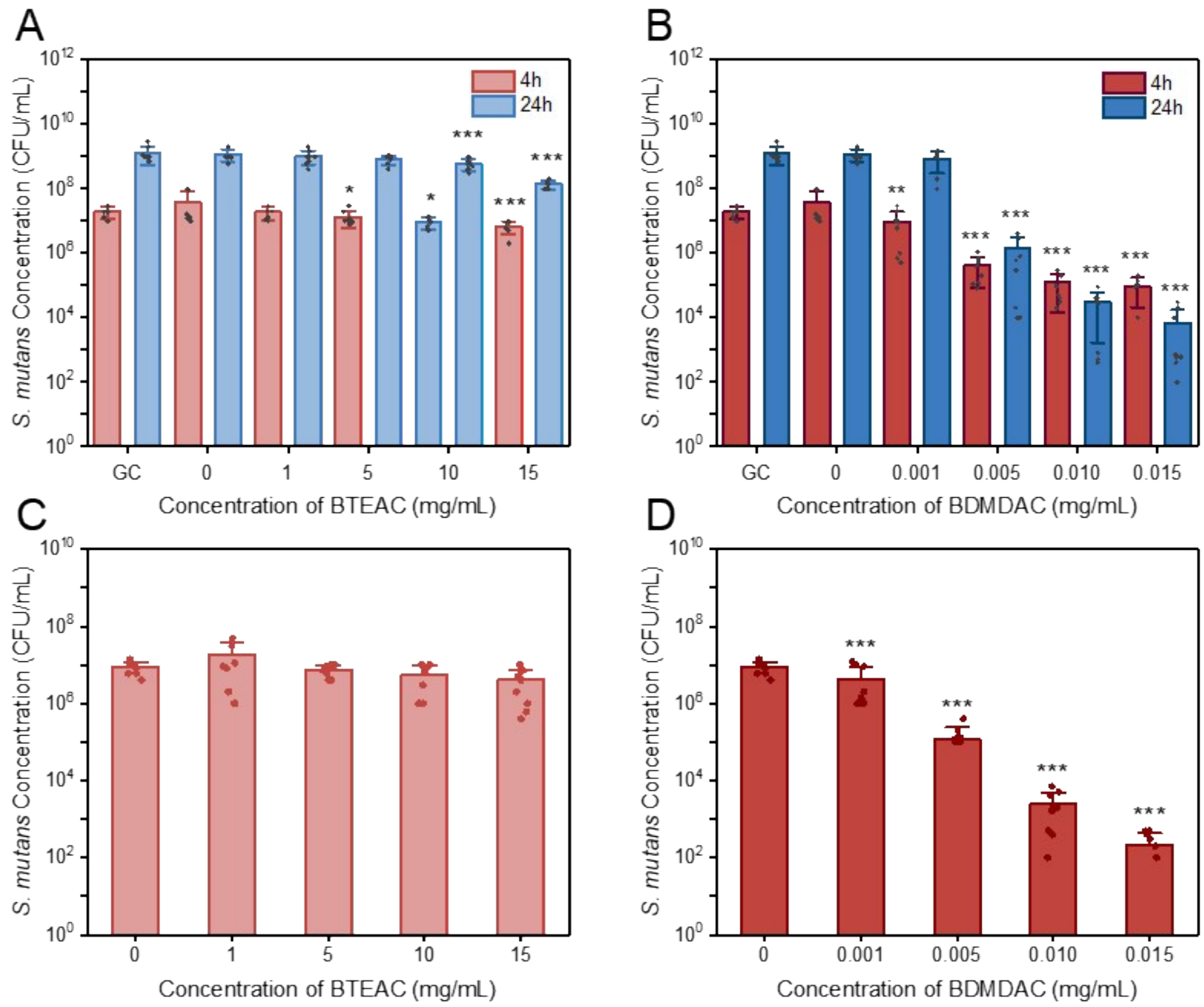

Figure S10. The antimicrobial activity of electrospun nanofibers against *S. mutans* under anaerobic conditions. (A) PVA-BTEAC and (B) PVA-BDMDAC nanofibers against planktonic bacteria after 4- and 24-hour incubation. Biofilm inhibition of (C) PVA-BTEAC and (B) PVA-BDMDAC nanofibers after 24-hour incubation. *$p < 0.05$, **$p < 0.01$, and ***$p < 0.001$ from the corresponding concentrations with control nanofibers.

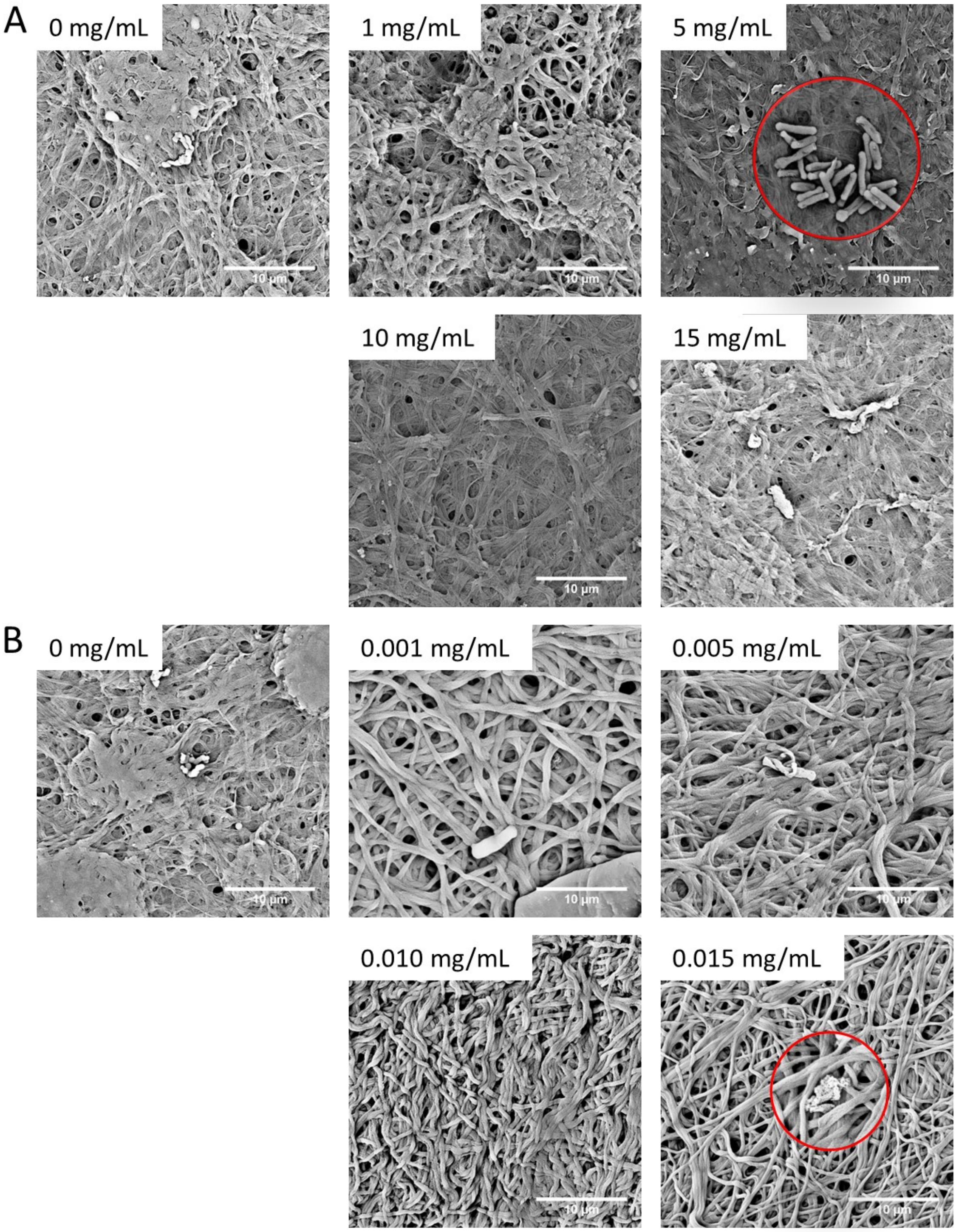

Figure S11. SEM images of 24 h *P. gingivalis* biofilm exposed to electrospun nanofibres. (A) PVA-BTEAC nanofibres surface at concentrations from 0 – 15 mg/mL; and (B) PVA-BDMDAC nanofibres surface at concentrations from 0 – 0.015 mg/mL. Scale bars = 10 µm.
